# Palmitate-induced lipotoxicity is crucial for the pathogenesis of nonalcoholic fatty liver disease in cooperation with gut-derived endotoxin

**DOI:** 10.1038/s41598-018-29735-6

**Published:** 2018-07-27

**Authors:** Yuji Ogawa, Kento Imajo, Yasushi Honda, Takaomi Kessoku, Wataru Tomeno, Shingo Kato, Koji Fujita, Masato Yoneda, Satoru Saito, Yusuke Saigusa, Hideyuki Hyogo, Yoshio Sumida, Yoshito Itoh, Kosei Eguchi, Takeharu Yamanaka, Koichiro Wada, Atsushi Nakajima

**Affiliations:** 1 Department of Gastroenterology and Hepatology, Yokohama City University Graduate School of Medicine, Yokohama, 236-0004 Japan; 2 Department of Biostatistics, Yokohama City University School of Medicine, Yokohama, 236-0004 Japan; 3 Department of Gastroenterology and Hepatology, JA Hiroshima General Hospital, Hiroshima, 738-8503 Japan; 4 Division of Hepatology and Pancreatology, Department of Internal Medicine, Aichi Medical University, Aichi, 480-1195 Japan; 5 Department of Gastroenterology and Hepatology, Kyoto Prefectural University of Medicine, Kyoto, 602-8566 Japan; 6 Department of Cardiovascular Medicine, Graduate School of Medicine, University of Tokyo, Bunkyo, 113-8655 Japan; 7 Department of Pharmacology, Shimane University Faculty of Medicine, Izumo, 693-8501 Japan

## Abstract

Although previous studies have indicated important roles of palmitate, a saturated fatty acid, in the pathogenesis of nonalcoholic fatty liver disease (NAFLD), it remains unclear how palmitate contributes to inflammation and fibrosis in the liver. Administration of palmitate in high fat diet (HFD)-fed but not basal diet (BD)-fed mice resulted in an increase in serum alanine aminotransferase (ALT) levels. Surprisingly, combined administration of very low dose lipopolysaccharide in palmitate-treated mice led to a marked increase in serum ALT levels despite BD-fed conditions. Administration of palmitate alone in BD-fed mice caused inflammatory cell infiltration and liver fibrosis mediated by the toll-like receptor 4 pathway without ALT elevation. In addition, a significant correlation between serum free fatty acid levels and liver fibrosis stage was observed in patients with NAFLD. These results indicate that palmitate may play crucial roles in the pathogenesis of NAFLD in the presence of gut-derived endotoxin.

## Introduction

Nonalcoholic fatty liver disease (NAFLD) is recognized as the most common liver disease associated with metabolic comorbidities, which include obesity, type 2 diabetes, hyperlipidemia, hypertension and metabolic syndrome^1^. The incidence of NAFLD is increasing, paralleled by the significant increase in obesity, and is an emerging health problem worldwide, affecting between 25% and 30% of the general population^2,3^.

NAFLD can be histologically classified into two subtypes, nonalcoholic fatty liver (NAFL) and nonalcoholic steatohepatitis (NASH). NAFL is defined as the presence of hepatic steatosis with no evidence of hepatocellular injury and is generally considered to be benign with negligible risk of progression to advanced fibrosis and liver-related mortality. NASH is defined by the presence of hepatic steatosis with evidence of hepatocyte damage and is generally considered to be progressive with substantial risk of progression to advanced fibrosis and liver-related mortality^1^. Recently, paired liver biopsy studies revealed that liver fibrosis progresses in patients with NAFLD (NAFL and NASH)^4^. However, the molecular mechanism underlying the progression of liver fibrosis in NAFL and NASH remains unclear.

Fatty liver is the result of excessive accumulation of various lipids, and triglycerides (TGs) are the most conspicuous type of lipids in fatty liver^5^. Free fatty acids (FFAs) contribute to the liver-TG pool, and the primary sources of FFAs are serum FFAs from adipose tissue and dietary fatty acids^6^.

Serum FFA levels are elevated in obese subjects^7^. We have previously reported that serum FFA levels are elevated in patients with NAFLD^8^. Almeida IT *et al*. showed that the proportion of palmitate in serum FFAs is elevated in patients with NASH^9^. Palmitate, a saturated FFA, is the most common circulating FFA. Therefore, the effects of palmitate on hepatocytes or the liver are attracting significant attention. An *in vitro* study showed that saturated fatty acids induced hepatocyte lipoapoptosis, and palmitate was more toxic than other saturated and unsaturated fatty acids^10^. An *in vivo* study also showed that inhibition of TG synthesis caused FFA accumulation in the liver, resulting in severe injury and fibrosis^11^. Therefore, FFAs, especially palmitate, are considered to be one of the most important factors that play a crucial role in the pathogenesis of NAFLD^12^. However, it remains unclear how palmitate contributes to inflammation and fibrosis in the liver and what molecular mechanisms are involved in the pathogenesis of NAFLD, progressed inflammation and fibrosis. Because palmitic acid alone has a weak effect based on preliminary study results, a further mechanism is assumed to exist. We, therefore, hypothesized that cooperative mechanisms in addition to palmitate might exist in the progression of NAFLD.

Endotoxin, a complex lipopolysaccharide (LPS), is a potent trigger of innate immunity and considered to be an influential key player in liver inflammation in NASH pathogenesis^13,14^. The elevated blood endotoxin level in obesity, which is accompanied by several metabolic disorders, is associated with low-grade systemic inflammation^15,16^. The fat- and/or fructose-rich Western-style diet contributes to elevated plasma endotoxin levels by causing changes in gastrointestinal barrier function or the microbiota composition and leads to the development of NAFLD^15,17^. Furthermore, we previously revealed that obesity-induced response to endotoxin plays a crucial role in NASH progression^13^. Therefore, it is thought that a cooperative mechanism between endotoxin and palmitate in the pathogenesis of NAFLD might be important.

In this study, we found that administration of palmitate in high fat diet (HFD)-fed but not basal diet (BD)-fed mice led to increased serum alanine aminotransferase (ALT) levels. In addition to the increase in ALT levels, HFD-fed mice exhibited severe fibrosis in the liver compared with mild fibrosis of BD-fed mice. Combined administration of LPS in palmitate-treated mice resulted in a marked increase in serum ALT levels despite BD-fed conditions. Our results revealed that palmitate-mediated molecular mechanisms cooperated with LPS in the pathogenesis of NAFLD.

## Results

### Palmitate induced neutrophil and macrophage infiltration in the liver

Three hours after a single intraperitoneal injection of emulsified ethyl palmitate, serum FFA levels were significantly increased compared with those of untreated or vehicle-treated mice both under BD-fed and HFD-fed conditions (Fig. S1A). These results indicate that 3 μmol/g palmitate (300 mM) is sufficient to increase serum FFA levels.

**Figure 1:**
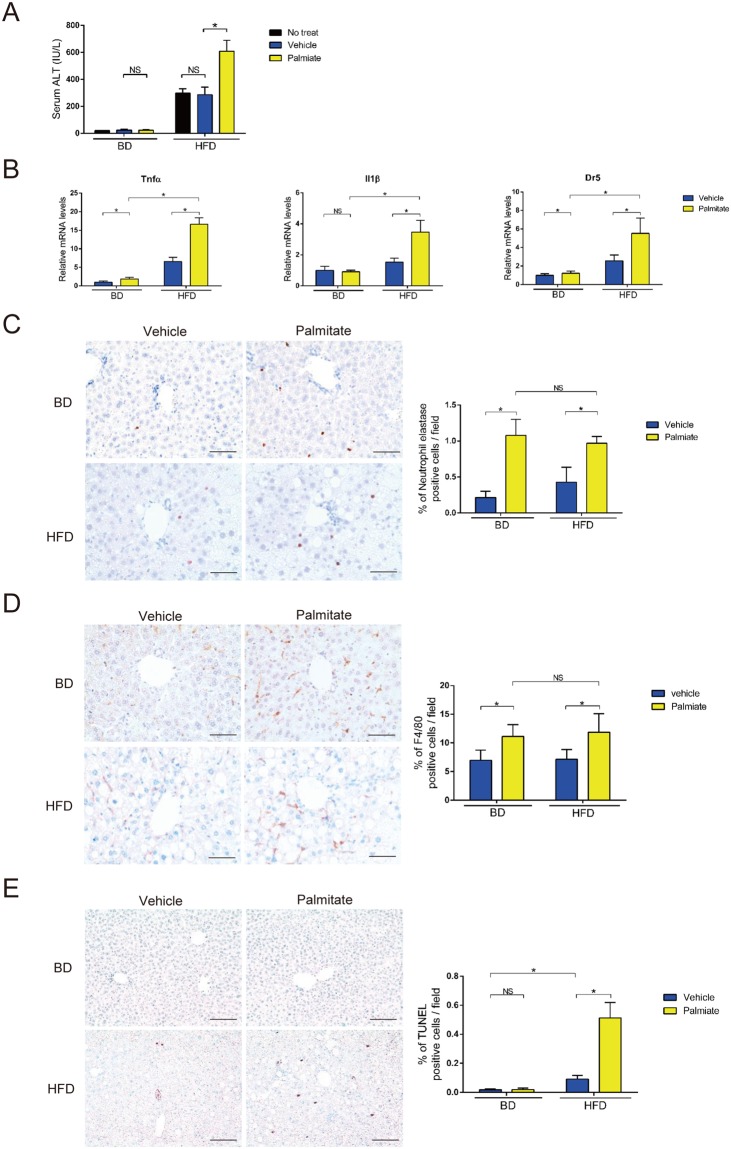
A single palmitate injection induced liver inflammation. (**A**) Serum ALT levels were measured 24 h after a single intraperitoneal injection of palmitate, vehicle or no treatment in BD- and HFD-fed 28-week-old WT mice. (**B**) Tnfα, IL1β and Dr5 mRNA levels in whole liver were measured by qPCR 3 h after a single intraperitoneal injection of palmitate or vehicle in BD- and HFD-fed 28-week-old WT mice. (**C**,**D**) Immunohistochemical detection (×400) of NE and F4/80 in representative liver samples and average % of NE- (×100) and F4/80-positive (×200) cells 3 h after palmitate or vehicle injection were examined in BD- and HFD-fed 28-week-old WT mice. Scale bars, 50 μm. (**E**) Immunohistochemical detection of TUNEL (×200) in representative liver samples and average % of TUNEL-positive cells (×100) 24 h after palmitate or vehicle injection were examined in BD- and HFD-fed 28-week-old WT mice. Scale bars, 100 μm. n = 5 mice per group. Data represent the mean ± SD. *P < 0.05.

We found that serum ALT levels in HFD-fed mice were elevated 24 h after single intraperitoneal administration of palmitate in a concentration-dependent manner (Fig. S1B). Administration of 3 μmol/g palmitate was the highest dose that elevated serum ALT levels in HFD-fed mice but not BD-fed mice (Figs 1A and S1B). Palmitate injection increased hepatic mRNA expression of proinflammatory cytokines (tumor necrosis factor α [TNFα] and interleukin-1β [IL-1β]) and TNF receptor family (death receptor 5 [DR5]). The elevations were higher in HFD-fed mice than BD-fed mice (Fig. 1B). Immunohistochemical staining revealed that palmitate injection increased infiltration of neutrophil elastase (NE) expressing neutrophils and F4/80 expressing macrophages in both BD-fed and HFD-fed mice (Fig. 1C,D). The numbers of infiltrated neutrophils and macrophages were highest 3 h after palmitate injection (Fig. S1C,D). In addition, palmitate induced more inflammatory cell infiltration than other fatty acids (Fig. S1E,F). Interestingly, the elevation in serum ALT levels was observed in HFD-fed but not BD-fed mice, although the number of infiltrated neutrophils and macrophages to hepatocytes was similar between BD-fed and HFD-fed mice (Fig. 1A–D). The extent of apoptosis detected by TUNEL assay and cleaved caspase 3 was increased in HFD-fed mice compared with that in BD-fed mice (Figs 1E and S1G).

These results indicate that increase in palmitate level under HFD-induced obese conditions might be a causative step in the increase in hepatic proinflammatory cytokine expression and apoptosis, resulting increased serum ALT. We, therefore, focused on the mechanisms underlying the palmitate-induced elevation of serum ALT levels under HFD conditions.

### Gut-derived endotoxin exacerbated palmitate-induced liver injury

**Figure 2:**
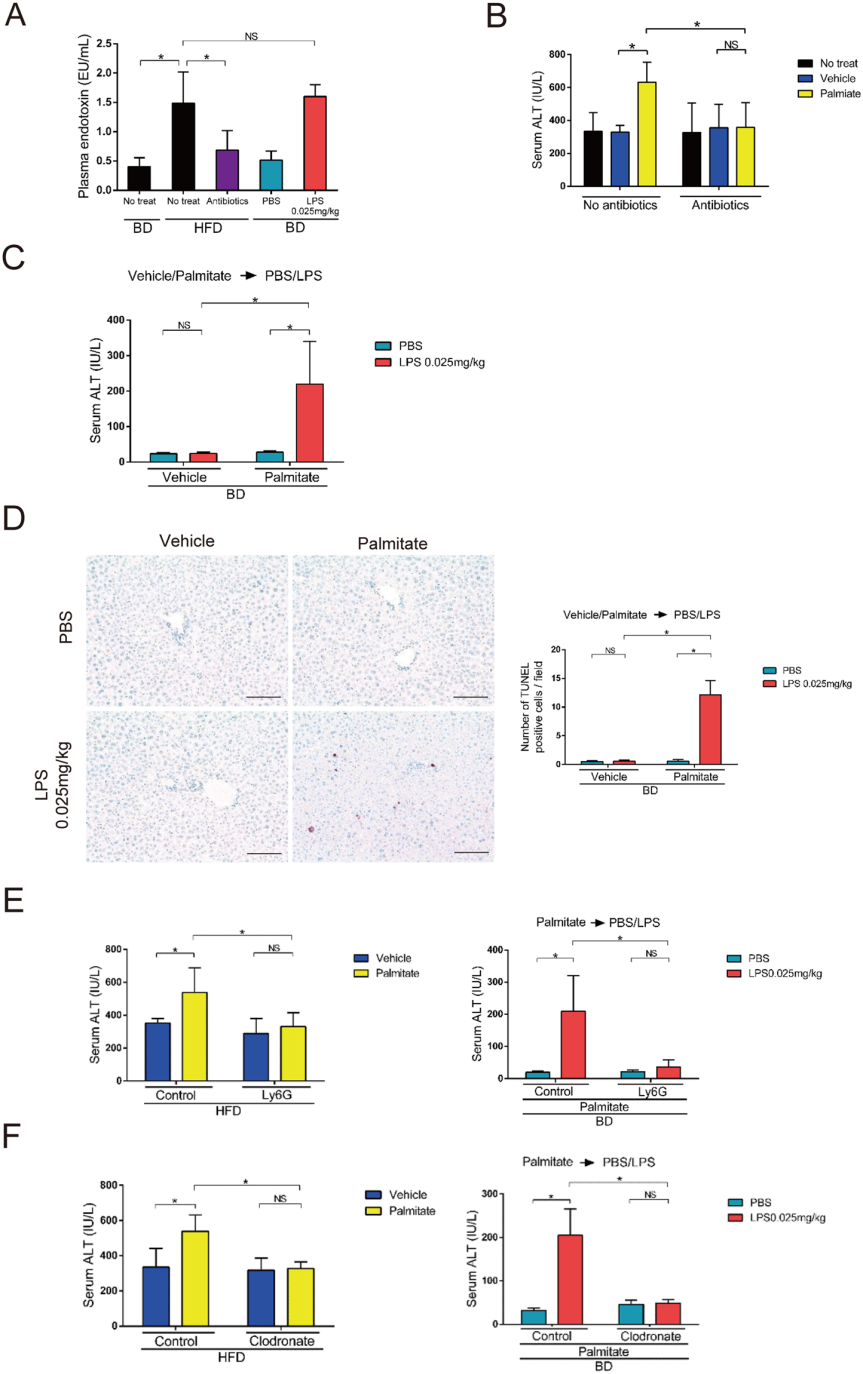
Palmitate and gut-derived endotoxin elevated serum ALT levels. (**A**) Plasma endotoxin levels were measured in 28-week-old BD-fed WT mice 24 h after a single intraperitoneal injection of 0.025 mg/kg LPS or PBS or no treatment and in HFD-fed 28-week-old WT mice 4 weeks after gut-sterilization or no treatment. (**B**) Serum ALT levels were measured 24 h after a single intraperitoneal injection of palmitate or vehicle or no treatment in HFD-fed 28-week-old mice that were gut-sterilized for 4 weeks or untreated. (**C**) Serum ALT levels were measured 24 h after 0.025 mg/kg LPS or PBS injection in BD-fed 28-week-old mice. LPS or PBS was injected 1 h after palmitate or vehicle injection. (**D**) Immunohistochemical detection of TUNEL (×200) in representative liver samples and average numbers of TUNEL-positive cells (×100) 24 h after 0.025 mg/kg LPS or PBS injection were examined in BD-fed 28-week-old WT mice. LPS or PBS was injected 1 h after palmitate or vehicle injection. Scale bars, 100 μm. (**E**) Ly-6G or control IgG was intraperitoneally injected 24 h prior to palmitate or vehicle injection in 28-week-old HFD-fed (Left) and BD-fed mice (Right). In HFD-fed mice, serum ALT levels were measured 24 h after palmitate or vehicle injection. In BD-fed mice, serum ALT levels were measured 24 h after 0.025 mg/kg LPS or PBS injection, which was performed 1 h after palmitate injection. (**F**) Clodronate or control liposomes were injected into the tail vein 48 h prior to palmitate or vehicle injection in 28-week-old HFD-fed (Left) and BD-fed mice (Right). In HFD-fed mice, serum ALT levels were measured 24 h after palmitate or vehicle injection. In BD-fed mice, serum ALT levels were measured 24 h after 0.025 mg/kg LPS or PBS injection, which was performed 1 h after palmitate injection. n = 5 mice per group. Data represent the mean ± SD. *P < 0.05. NS, not significant.

Elevation of blood endotoxin levels, which occurs in obesity, is associated with low-grade systemic inflammation^15,16^. We found that plasma endotoxin levels were higher in HFD- than BD-fed mice (Figs 2A and S2K). Next, we found that 4-week treatment with a cocktail of broad-spectrum antibiotics (ampicillin, neomycin, metronidazole and vancomycin) efficiently reduced plasma endotoxin levels in HFD-fed mice to levels comparable with those in BD-fed mice (Fig. 2A). Furthermore, palmitate injection did not elevate serum ALT levels in gut-sterilized HFD-fed mice (Fig. 2B). Thus, the presence of gut-derived endotoxin is required for palmitate-induced serum ALT elevation.

We next intraperitoneally administered purified LPS, a major outer membrane constituent of gram-negative bacteria. We selected 0.025 mg/kg LPS (a very low dose) for subsequent experiments, because this dose without palmitate was the highest that did not significantly increase serum ALT levels in BD-fed mice (Figs 2C and S2C). Plasma endotoxin levels were similar between non-LPS treated BD-fed mice and PBS-treated BD-fed mice. Additionally, plasma endotoxin levels were similar between 0.025 mg/kg LPS-treated BD-fed mice and no-treated HFD-fed mice (Fig. 2A). We then intraperitoneally injected LPS 1 h after palmitate or vehicle injection in BD-fed mice to investigate the response against 0.025 mg/kg LPS. Palmitate plus LPS injection increased serum ALT levels compared with those following vehicle-LPS or palmitate-PBS injection (Fig. 2C). However, the number of neutrophils and macrophages were similar between palmitate plus LPS-injected mice and palmitate plus PBS-injected mice (Fig. S2E,F). The number of apoptotic cells detected by TUNEL and cleaved caspase 3 was higher in palmitate-LPS-injected mice than in palmitate-PBS-injected mice (Figs 2D and S2G). Serum ALT levels were also elevated by intraperitoneal palmitate injection 1 h after LPS injection in BD-fed mice (Fig. S2D). These results suggest that the plasma endotoxin elevation induced by very-low-dose LPS is indispensable for palmitate-induced apoptosis in the liver and serum ALT elevation.

We next assessed the role of neutrophils and macrophages in serum ALT elevation. Ly-6G antibody decreased palmitate-induced neutrophil infiltration in the liver (Fig. S2H). Ly-6G antibody administration significantly suppressed the palmitate-induced serum ALT elevation in HFD-fed mice (Fig. 2E) and the palmitate plus LPS–induced serum ALT elevation in BD-fed mice (Fig. 2F). Next, we found that clodronate liposomes effectively depleted macrophages in the liver (Fig. S2I,J). Similarly to Ly6G antibody treatment, clodronate liposomes significantly suppressed the palmitate-induced serum ALT elevation in HFD-fed mice (Fig. 2G) and the palmitate plus LPS–induced serum ALT elevation in BD-fed mice (Fig. 2G). These results suggest that both neutrophil and macrophage infiltrations induced by palmitate are indispensable for the response to LPS resulting in the elevation of serum ALT levels.

### Palmitate induced neutrophil and macrophage infiltrations in the liver via the toll-like receptor 4 (TLR4) pathway

**Figure 3:**
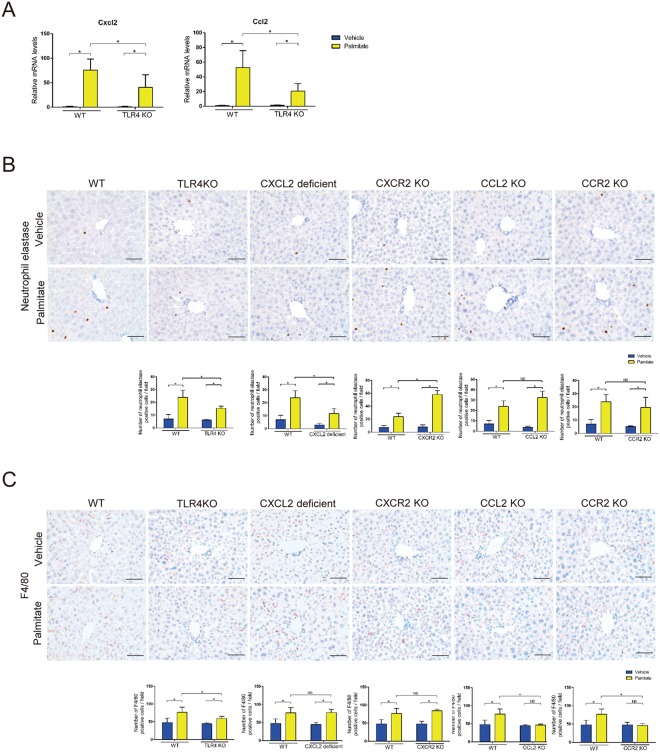
Palmitate increased liver inflammation by chemokines via the TLR4 pathway. (**A**) Cxcl2 and Ccl2 mRNA levels in whole liver were analyzed by qPCR 3 h after palmitate or vehicle injection in BD-fed 8-week-old WT and TLR4 KO mice. (**B**,**C**) Immunohistochemical detection of NE and F4/80 (×400) in representative liver samples and average numbers of NE- (×100) and F4/80-positive (×200) cells 3 h after palmitate or vehicle injection were examined in BD-fed 8-week-old WT, TLR4 KO, CXCL2 deficient, CXCR2 KO, CCL2 KO and CCR2 KO mice. n = 5 mice per group. Data represent the mean ± SD. *P < 0.05. NS, not significant.

cDNA microarray analysis of whole livers after a single palmitate injection in BD-fed mice revealed that palmitate induced the expression of genes encoding chemokines (Table S2). Real-time PCR analysis confirmed these findings (Figs 3A and S3A). Cxcl2 contributes to neutrophil infiltration in the tissue^18^, and Ccl2 contributes to macrophage infiltration^19^. We identified the cells that produced Cxcl2 or Ccl2. By *in situ* hybridization, Cxcl2 mRNA was detected in parenchymal cells, while Ccl2 mRNA was detected in non-parenchymal cells in the liver (Fig. S3B).

Recently, many studies have suggested that the pathway involving TLR4, which is a pattern recognition receptor that initiates innate immune responses, plays a key role in lipotoxicity^20–22^. In the present study, TLR4 knockout (KO) mice displayed a significant reduction in hepatic Cxcl2 and Ccl2 mRNA expression, but not Cxcl1, Cxcl10, and Ccl3 mRNA expression, compared with WT mice. In addition, TLR4 KO mice exhibited a significant reduction in neutrophil and macrophage infiltrations in the liver compared with WT mice (Figs 3A–C and S3A). These results indicate that palmitate-induced neutrophil and macrophage infiltrations in the liver are due to chemokines (Cxcl2 and Ccl2) via the TLR4 pathway.

C-X-C motif chemokine receptor (CXCR2) is the gene encoding the CXCL2 receptor, and C-C motif chemokine receptor (CCR2) is the gene encoding the CCL2 receptor. We found that palmitate did not increase neutrophil infiltration in the livers of CXCL2 deficient mice or macrophage infiltration in the livers of CCL2 and CCR2 KO mice (Fig. 3B,C). These data suggest that CXCL2 is important for neutrophil infiltration, and CCL2 and CCR2 are important for macrophage infiltration in the liver in response to palmitate. Neutrophil infiltration in the liver was not affected in CXCR2 KO mice, which suggests that a compensatory mechanism for neutrophil recruitment is active in these mice.

Various fatty acid species exert different effects on cellular function. For example, monounsaturated fatty acids induce significant TG formation but do not initiate apoptosis^23,24^. In the present study, we found that saturated fatty acids, particularly palmitate, induced neutrophil and macrophage infiltrations in the liver because of increased Cxcl2 and Ccl2 mRNA expression compared with monounsaturated fatty acids (Figs S1E,F and S3C,D). These results suggest that palmitate induces liver inflammation more effectively than other fatty acids.

### Chronic exposure to palmitate induced mild liver fibrosis

**Figure 4:**
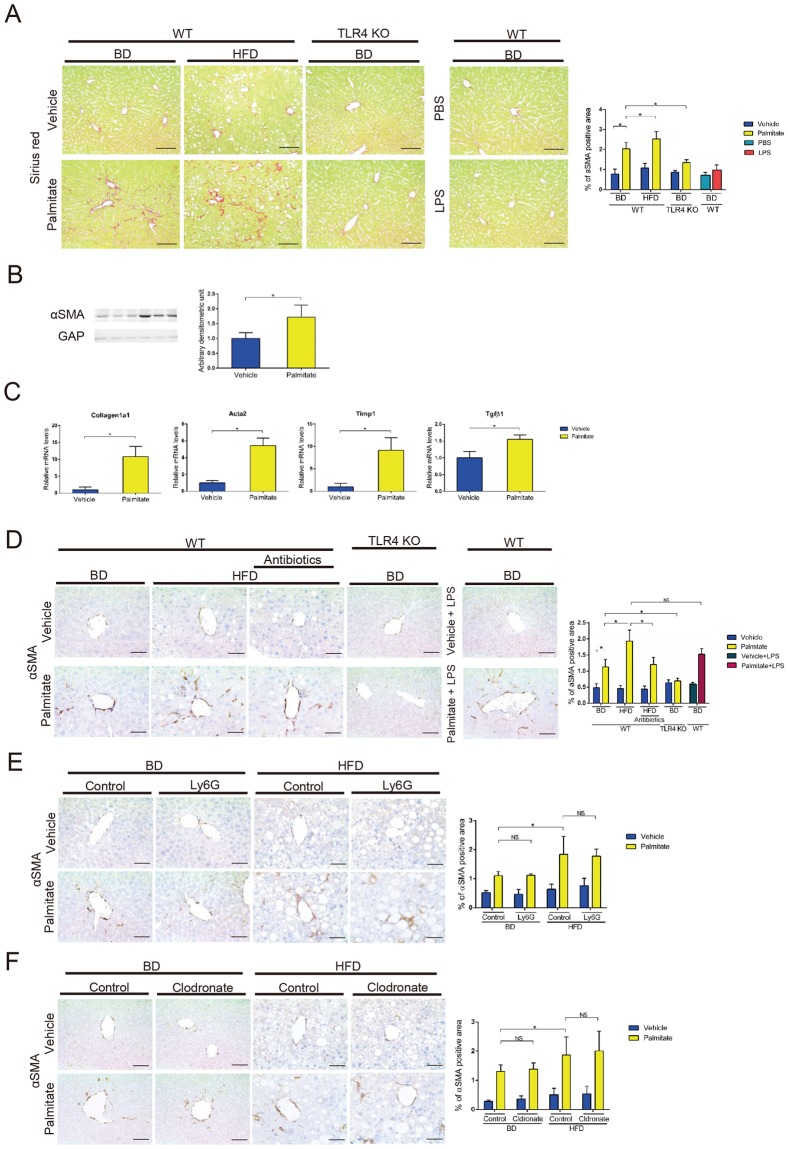
Long-term palmitate injection induced liver fibrosis. (**A**–**C**) We intraperitoneally injected 3 μmol/g palmitate, vehicle, 0.025 mg/kg LPS or PBS twice a day for 2 weeks into 8-week-old *ad libitum* BD-fed WT mice, 28-week-old *ad libitum* HFD-fed WT mice and 8-week-old *ad libitum* BD-fed TLR4 KO mice. (**A**) Collagen deposition was evaluated by sirius red staining. Immunohistochemical detection of sirius red (×200) in representative liver and quantification of sirius red staining (×100) in liver were analyzed in long term palmitate or vehicle injected 10-week-old BD-fed WT mice, 10-week-old BD-fed TLR4 KO mice and 30-week-old HFD-fed WT mice. Scale bars, 100 μm. n = 5 mice per group. (**B**) αSMA expression in whole liver was determined by western blot analysis in long term palmitate or vehicle injected BD-fed 10-week-old WT mice. n = 3 mice per group. (**C**) Acta2, tissue inhibitor of metalloproteinases-1 (Timp1), collagen1a1 and transforming growth factor beta 1 (Tgfβ1) mRNA expression levels in whole liver were analyzed by qPCR in long term palmitate or vehicle injected BD-fed 10-week-old WT mice. n = 5 mice per group. (**D**–**F**) A single palmitate injection induced HSC activation. A single intraperitoneal injection of 3 μmol/g palmitate, vehicle, pamitate plus 0.025 mg/kg LPS, vehicle plus 0.025 mg/kg LPS was administered to the mice. Scale bars, 50 μm. n = 5 mice per group. (**D**) Immunohistochemical detection of αSMA (×400) in representative liver samples and average % of αSMA-positive area (×100) 24 h after palmitate, vehicle, vehicle-0.025 mg/kg LPS or palmitate-0.025 mg/kg LPS injection were examined in 8-week-old BD-fed WT mice, 8-week-old BD-fed TLR4 KO mice and 28-week-old HFD-fed mice that were gut-sterilized for 4 weeks or untreated. (**E**) Ly-6G or control IgG was intraperitoneally injected. Immunohistochemical detection of αSMA (×400) in representative liver samples and average % of αSMA-positive area (×100) 24 h after palmitate or vehicle injection were examined in 8-week-old BD-fed and 28-week-old HFD-fed mice. (**F**) Clodronate or control liposomes were injected into the tail vein. Immunohistochemical detection of αSMA (×400) in representative liver samples and average % of αSMA-positive area (×100) 24 h after palmitate or vehicle injection were examined in 8-week-old BD-fed and 28-week-old HFD-fed mice. Data represent the mean ± SD. *P < 0.05. NS, not significant.

Palmitate injection for 2 weeks did not alter body weight except in HFD-fed mice, and 0.025 mg/kg LPS injection for 2 weeks did not alter body weight in BD-fed mice (Fig. S4A–D). After long-term palmitate injection, the BD-fed mice showed mild liver fibrosis by sirius red staining and alpha-smooth muscle actin (αSMA, encoded by Acta2) expression (Fig. 4A,B). Hepatic mRNA expression levels of early fibrogenesis markers, were elevated (Fig. 4C). Neutrophils and macrophages were infiltrated in the liver, but serum ALT levels were not significantly elevated (Fig. S4E–G). Interestingly, only one saturated fatty acids (palmitate) induced mild liver fibrosis in BD-fed WT mice, while very-low-dose LPS did not (Fig. 4A). Furthermore, long-term palmitate injection induced significantly more liver fibrosis in HFD- than BD-fed WT mice (Fig. 4A). These results suggest that HFD-induced obesity, in which plasma endotoxin levels are elevated, is important for palmitate-induced liver fibrosis. Coexistence of palmitate and very-low-dose LPS exacerbates liver fibrosis.

### The TLR4 pathway enhanced the fibrosis signal induced by palmitate

Hepatic stellate cells (HSCs) are considered the main fibrogenic cell type of the liver. HSC activation leads to increased expression of contractile filaments, such as αSMA^25,26^. We found that after a single palmitate injection the αSMA-positive area in the liver increased in a time-dependent manner (Fig. S5A), suggesting a correlation between palmitate and liver fibrosis. Additionally, long-term palmitate injection enhanced αSMA expression, accompanied by liver fibrosis (Fig. 4A–C).

We examined the effect of obesity and found that the αSMA-positive area 24 h after a single palmitate injection was enhanced in HFD-fed mice compared with BD-fed mice, while gut-sterilization in HFD-fed mice reduced palmitate-induced HSC activation (Fig. 4D). Furthermore, palmitate plus LPS injection increased αSMA-positive area more than palmitate injection alone (Fig. 4D). Thus, these data confirm that cooperation between palmitate and endotoxin/LPS aggravates palmitate-induced HSC activation.

Next, after intraperitoneal injection of palmitate, TLR4 KO mice displayed a significant reduction in hepatic αSMA enhancement compared with WT mice (Fig. 4D). Furthermore long-term palmitate injection did not induce liver fibrosis in TLR4 KO mice. This indicates that palmitate activates HSCs via the TLR4 pathway. On the other hand, the αSMA-positive area was upregulated by a single palmitate injection in CCL2 KO, CCR2 KO, CXCL2 deficient and CXCR2 KO mice (Fig. S5B).

We decreased the number of neutrophils using Ly-6G antibody and found that the αSMA-positive area upregulated by palmitate injection was similar between the Ly-6G-treated mice and the control IgG-treated mice following BD and HFD feeding (Fig. 4E). Next, we depleted the number of macrophages using clodronate liposomes and found that the αSMA-positive area upregulated by palmitate injection was similar between the clodronate and control liposome-treated mice following BD and HFD feeding (Fig. 4F). These results suggest that HSCs are activated by palmitate without macrophages or neutrophils *in vivo*.

### Serum FFA levels and whole blood endotoxin correlated with liver fibrosis and serum ALT levels in patients with NAFLD

**Figure 5:**
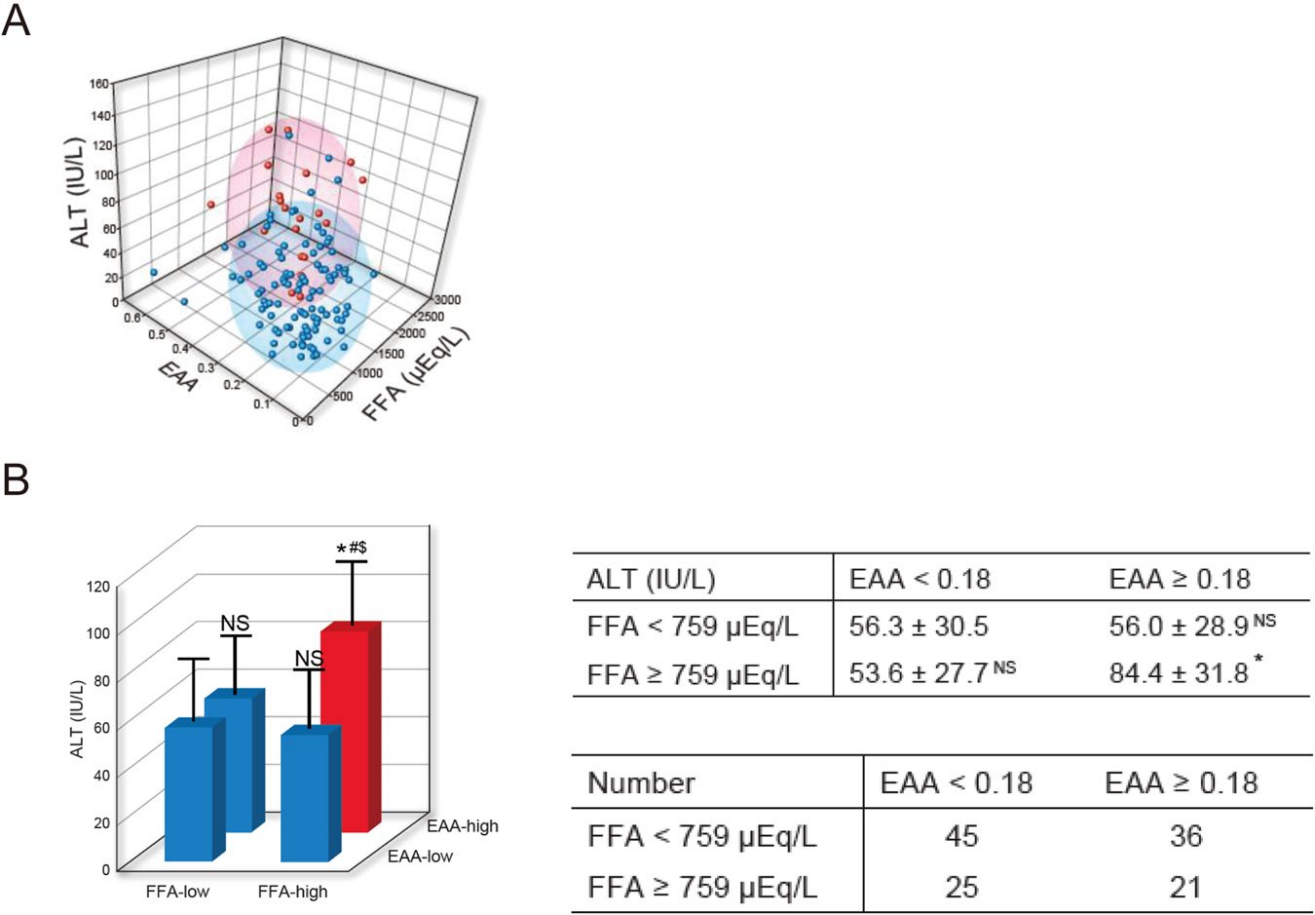
Serum ALT levels of “FFA-high and EAA-high” patients showed high serum ALT. Correlations between serum FFA levels, whole blood EAA and serum ALT levels were analyzed in 127 patients with NAFLD (fibrosis stages 0, 1 and 2). (**A**) Serum FFA, whole blood EAA and serum ALT were plotted in three dimensions. “FFA-high and EAA-high” group is red. “FFA-low and EAA-low”, “FFA-low and EAA-high” and “FFA-high and EAA-low” groups are blue. Ellipsoid means cumulative probability = 0.75. (**B**) Serum ALT levels of “FFA-low and EAA-low (n = 45)”, “FFA-low and EAA-high (n = 36)”, “FFA-high and EAA-low (n = 25)” and “FFA-high and EAA-high (n = 21)” groups were analyzed. Results are presented as means ± SD. *P < 0.05 compared with “FFA-low, EAA-low”. ^#^P < 0.05 compared with “FFA-low, EAA-high”. ^$^P < 0.05 compared with “FFA-high, EAA-low”. NS, not significant compared with “FFA-low, EAA-low”.

Table S4 shows the characteristics of patients with NAFLD (fibrosis stages 0, 1 and 2) examined in this study. These patients’ average serum FFA was 759 μEq/L and average whole blood endotoxin activity assay (EAA) was 0.18. Thus, we created four subgroups: “FFA-low” (serum FFA < 759 μEQ/L), “FFA-high” (serum FFA ≥ 759 μEQ/L), “EAA-low” (whole blood EAA < 0.18), and “EAA-high” (whole blood EAA ≥ 0.18). We plotted serum FFA, whole blood EAA and serum ALT in three dimensions (Fig. 5A). Serum ALT levels of “FFA-high and EAA-high” patients were significantly higher than those of “FFA-low and EAA-low”, “FFA-low and EAA-high” and “FFA-high and EAA-low” patients (Fig. 5A,B, table S5). The Multiple linear regression analysis for serum ALT levels using the serum FFA levels, EAA, the serum FFA levels x EAA, age, platelet count, and body mass index identified that the serum FFA levels x EAA, age, and body mass index are significantly and independently associated with serum ALT levels (Table S6). These results suggest that cooperation between serum FFA and whole blood endotoxin aggravates serum ALT levels.

Table S8 shows the characteristics of patients with NAFLD examined in this study. The platelet count, aspartate aminotransferase (AST), type 4 collagen and hyaluronic acid levels were positively correlated with the liver fibrosis stage in patients with NAFLD (Table 1). Serum FFA levels and liver fibrosis displayed a modest positive correlation (r = 0.204, P < 0.001) (Table 1). The Multiple linear regression analysis for liver fibrosis stage using body mass index, platelet count, serum levels of AST, triglyceride, FFA, and type IV collagen 7 S identified that they are significantly and independently associated with liver fibrosis stage (Table S9). These results suggest that serum FFAs promote liver fibrosis in patients with NAFLD.

**Table 1:** Correlations between histopathologic features of liver biopsies and clinical parameters in patients with NAFLD.

N = 498		steatosis	Lobular inflammation	ballooning	fibrosis
Body mass index	r	0.148	0.099	0.122	0.116
	p	0.001	0.028	0.007	0.010
Platelet count	r	0.137	−0.047	−0.009	−0.235
	p	0.002	0.292	0.841	1.12 × 10^−7^
AST	r	0.198	0.346	0.271	0.348
	p	8.92 × 10^−6^	1.71 × 10^−15^	7.53 × 10^−10^	1.19 × 10^−15^
ALT	r	0.302	0.270	0.250	0.117
	p	5.52 × 10^−12^	9.07 × 10^−10^	1.55 × 10^−8^	0.009
Total cholesterol	r	0.057	0.025	0.029	−0.141
	p	0.207	0.576	0.514	0.002
Triglyceride	r	0.161	−0.040	0.057	−0.159
	p	3.18 × 10^−4^	0.372	0.208	3.53 × 10^−4^
HDL cholesterol	r	−0.078	0.019	0.002	0.041
	p	0.081	0.669	0.957	0.362
LDL cholesterol	r	0.038	0.032	0.055	−0.114
	p	0.394	0.470	0.217	0.011
Free fatty acid	r	−0.086	0.077	0.017	0.204
	p	0.054	0.085	0.708	4.60 × 10^−6^
Type IV collagen 7S	r	0.142	0.244	0.244	0.386
	p	0.002	3.31 × 10^−8^	3.42 × 10^−8^	3.72 × 10^−19^
Hyaluronic acid	r	−0.080	0.116	0.098	0.309
	p	0.073	0.009	0.029	1.76 × 10^−12^

Spearman’s rank correlation coefficient.

AST, aspartate aminotransferase; ALT, alanine aminotransferase; HDL, high-density lipoprotein; LDL, low-density lipoprotein.

Results are presented as means ± SD.

P < 0.05 for comparisons between groups using Spearman’s rank correlation coefficient.

## Discussion

**Figure 6:**
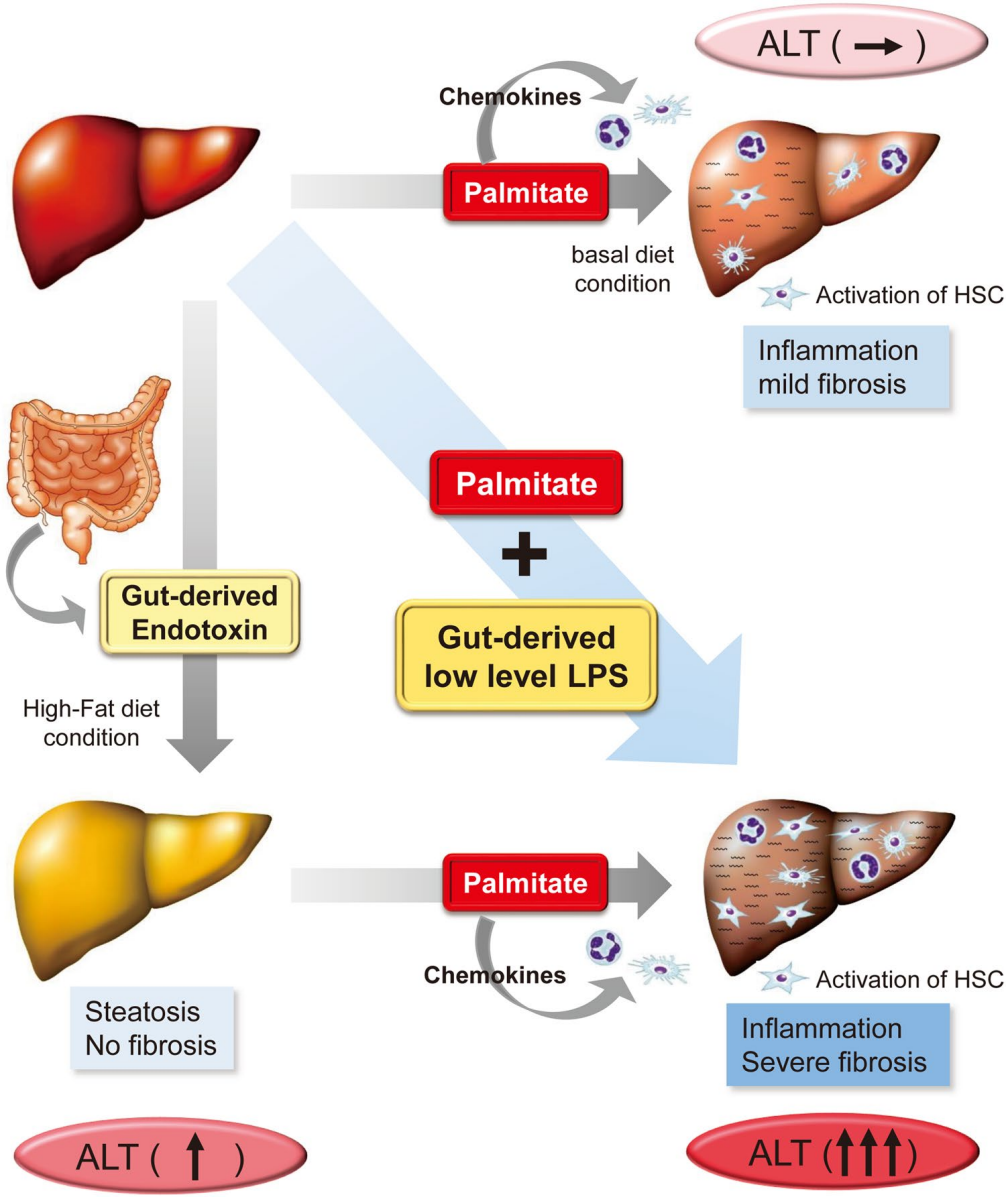
A schematic diagram showing the link between palmitate-induced lipotoxicity and endotoxine in NASH pathogenesis. Palmitate increased serum ALT levels in HFD-fed but not BD-fed mice. Palmitate induced inflammatory cell infiltration, HSC activation and liver fibrosis. Palmitate exacerbated NAFLD pathogenesis by cooperative interaction with gut-derived endotoxin.

In this study, we showed that (1) palmitate, a saturated fatty acid, elevated serum ALT levels in the presence of gut-derived endotoxin, (2) palmitate induced inflammatory cell infiltration in the liver by up-regulation of chemokines and stimulated mild liver fibrosis via the TLR4 pathway despite the absence of endotoxin, (3) palmitate lipotoxicity cooperated with gut-derived endotoxin in liver inflammation, and (4) fibrosis was also confirmed in patients with NAFLD (Fig. 6).

In our study, palmitate administration in HFD-fed but not BD-fed mice increased serum ALT levels. We found that plasma endotoxin levels in HFD-fed mice were significantly higher than those in BD-fed mice. Furthermore, gut sterilization reduced plasma endotoxin levels despite HFD consumption. These results indicate that gut microbiota effect a change in plasma endotoxin, and that HFD feeding may increase the permeability of gut endotoxin. Because the palmitate-induced serum ALT elevation was not observed in gut-sterilized HFD-fed mice, we propose that gut-derived endotoxin is the most important factor for palmitate-induced elevation of serum ALT levels in HFD-fed mice. We previously reported that hyper response to low-dose LPS (0.25 mg/kg) is important for inflammation and fibrosis in mouse models and patients with NAFLD^13^. In the present study, we confirmed that very-low-dose LPS (0.025 mg/kg) injection in BD-fed mice resulted in similar plasma endotoxin levels to those in HFD-fed mice. Therefore, we co-administered palmitate and very low dose LPS to BD-fed mice and revealed that palmitate in the presence of endotoxin significantly elevated serum ALT levels compared with those in the absence of endotoxin. These results clearly indicate that cooperation of palmitate and gut-derived endotoxin plays a crucial role in the elevation of serum ALT levels, an index of liver inflammation.

In patients with NAFLD, we confirmed that the marked elevation of serum ALT levels was observed in cases of high serum FFAs accompanied by high blood endotoxin compared with those of low serum FFAs with low blood endotoxin or high serum FFAs with low blood endotoxin or low serum FFAs with high blood endotoxin. In fact, several reports have shown that gut permeability, or small intestinal bacterial overgrowth, was observed more frequently in patients with NASH compared with healthy controls^27,28^. In addition, elevated plasma endotoxin levels were reported in subjects with NASH compared with healthy controls^29^. We also showed that enhanced response to endotoxin derived from bacteria is one of the key mechanisms underlying NASH progression^13^. Because palmitate is considered a major component of circulating saturated FFAs, the interaction between palmitate and endotoxin is important for elevation of serum ALT levels in patients with NAFLD. Limitation of this study is that we used different endotoxin assays for mice and human studies. Therefore the same assay is needed in the next study.

Pro-apoptotic DR5 mRNA-induced upregulation and apoptosis of inflammatory cytokine (TNFα and Il1β) were observed in palmitate-administered HFD-fed mice compared with BD-fed mice. Up-regulation of those molecules may induce liver inflammation, resulting in serum ALT elevation in HFD-fed mice. Single palmitate injection increased TNFα mRNA but not IL-1β mRNA in BD-fed mice, but long-term palmitate injection increased both TNFα and IL-1β mRNA (data not shown).

Csak T *et al*. showed that the saturated fatty acid, palmitate, induces sensitization to LPS-induced IL-1β release in mouse hepatocytes^30^. Malhi H *et al*. showed that FFAs directly affect sensitization of hepatocytes to tumor necrosis factor-related apoptosis inducing ligand-induced apoptosis by DR5 up-regulation. Specifically, enhanced DR5 expression was observed in liver biopsy samples from patients with NASH^31^. The palmitate-mediated up-regulation of inflammatory cytokines and induction of apoptosis in hepatocytes may be an important step in the development of liver inflammation and fibrosis.

A prominent feature of the inflammation observed in NASH is neutrophil accumulation^32,33^ and macrophage infiltration^34^ in the liver, resulting in the promotion of NASH. TLR4 activation increases the production of several proinflammatory cytokines^35^. It is well-known that TLR4 acts as a receptor for LPS^35^. Recently, several studies have demonstrated that TLR4 signaling is important for HFD-induced insulin resistance by mediating inflammatory responses within adipose tissue and skeletal muscle^22,36^. *In vivo*, the TLR4 pathway is essential for palmitate-induced inflammatory phenotypes in islet inflammation and neointima formation^20,21^. These studies suggest that the TLR4 pathway might be crucial for lipotoxicity in NASH pathogenesis. However, the molecular mechanisms underlying the induction of liver inflammation for each FFA remain poorly understood. In this study, we showed that palmitate induced neutrophil infiltration in the liver because of the expression of chemokines, primarily Cxcl2. Furthermore, palmitate stimulated macrophage infiltration through the expression of chemokines, primarily Ccl2, via the TLR4 pathway. In addition, we confirmed that palmitate induced neutrophil and macrophage infiltrations in the liver by stimulating much higher Cxcl2 and Ccl2 mRNA levels than other unsaturated fatty acids, such as palmitoleate (C8:1) and oleate (C18:1). We also found that Cxcl2 is important for neutrophil infiltration, and Ccl2/Ccr2 is important for macrophage infiltration in response to palmitate. These results indicate that palmitate can induce inflammatory cell infiltration in the liver mediated by up-regulation of various chemokines via the TLR4 pathway.

Liver fibrosis is associated with long-term outcomes in patients with NAFLD^37^. The TLR4 pathway also plays important roles in liver fibrosis^38,39^. However, the direct relationship between palmitate and the TLR4 pathway in liver fibrosis has not been investigated. In this study, we showed that long-term administration of palmitate induced mild liver fibrosis in a mouse model. The induction of mild liver fibrosis was due to activation of the TLR4 pathway. These results indicate that the TLR4 pathway is important for not only liver inflammation but also liver fibrosis in the pathogenesis of NAFLD.

HSCs are a primary cell type responsible for liver fibrosis following activation into fibrogenic myofibroblast-like cells^25,26^. Decreased neutrophils using Ly6G and depletion of macrophages using clodronate liposomes did not inhibit palmitate-induced HSC activation *in vivo* in an animal model. In our animal model, palmitate-induced HSC activation was not affected in CXCL2 deficient, CXCR2 KO, CCL2 KO or CCR2 KO mice. We could not show the mechanism by which palmitate induced αSMA expression. Further investigations are required to clarify the exact pathways impacting palmitate-induced HSC activation.

In this study, we determined that long-term palmitate administration stimulated HSC activation, resulting in mild liver fibrosis without ALT elevation in a mouse model. We previously reported that patients with NAFLD with ALT ranges that were almost normal (≤40) showed mild fibrosis. Specifically, 27.7% of patients were fibrosis stage 1, and 17.4% were fibrosis stage 2^40^. In this study, we also observed that serum FFA levels and liver fibrosis stage in patients with NAFLD were significantly correlated. Nehra V *et al*. showed that FFAs are elevated in patients with metabolic syndrome and correlated with fibrosis in patients with NAFLD^41^.

Administration of palmitate enhanced αSMA expression in HFD-fed mice compared with that in BD-fed mice. In fact, long-term palmitate administration in HFD-fed mice caused more severe fibrosis than that in BD-fed mice. The palmitate-induced aggravation of fibrosis in HFD-fed mice was suppressed by gut sterilization. These results indicate that the interaction between palmitate and the HFD-induced elevation of endotoxin is important for aggravation of liver fibrosis.

In conclusion, palmitate, a saturated fatty acid, induced serum ALT levels in the presence of gut-derived endotoxin. The effects of palmitate, such as induction of inflammatory cell infiltration in the liver, are important for the elevation of serum ALT levels. Palmitate also induced mild liver fibrosis. Palmitate lipotoxicity cooperated with gut-derived endotoxin in liver inflammation, and fibrosis was also confirmed in patients with NAFLD. Restricting palmitate intake and reducing gut-derived endotoxin could improve NAFLD pathogenesis. Further studies are necessary to confirm the molecular interaction between palmitate and endotoxin and the mechanism underlying gut-derived endotoxin elevation.

## Materials/Patients and Methods

### Studies of mouse

#### Animals and diets

C57BL/6 male mice (wild type [WT]) were purchased from CLEA Japan. TLR4 KO mice were purchased from Oriental Bio Service. CCL2 KO, CCR2 KO and CXCR2 +/− mice were purchased from Jackson Laboratory. CXCL2 +/− mice were generated by CRISPR/Cas9, and CXCL2 deficient mice were generated by crossing CXCL2 +/− mice. Mice were fed a BD (22% kcal from protein, 6% kcal from fat and 47% kcal from carbohydrates). For obesity-related studies, BD-fed mice were switched to a HFD (20% kcal from protein, 60% kcal from fat and 20% kcal from carbohydrates) at 8 weeks of age, and mice were fed a HFD for 20 weeks. Characteristics of these mice are shown in Table S1, 3. Mice were housed in a controlled environment (5 mice per cage, 12-h/12-h light and dark cycles) with free access to food and water. HFD-fed mice presented with steatosis without fibrosis. Body weights were regularly measured. All experiments with mice were conducted according to the Guidelines for Proper Conduct of Animal Experiments, Science Council of Japan, and protocols were approved by the Animal Care and Use Committee, Yokohama City University Medical School.

#### Palmitate and/or LPS injection

Ethyl palmitate has been shown to be rapidly hydrolyzed to free palmitate in the blood of rodents^20,21,42^. To assess the physiological role of FFAs *in vivo*, Shen H *et al*. established a novel method to specifically increase palmitate levels in the circulation of mice^21^. We used the same method to investigate the effect of FFAs on mouse livers. Ethyl palmitate (Tokyo Chemical Industry) was dissolved with 4.8% lecithin (Wako) and 10% glycerol in water to produce a mixture containing various concentrations of ethyl palmitate (50 mM, 100 mM, 150 mM, 300 mM and 600 mM), 1.2% lecithin and 2.5% glycerol. The mixture was then emulsified using a sonicator. The lecithin-glycerol-water solution was used as the vehicle. To investigate response against a single palmitate injection, we intraperitoneally injected 0.01 mL/g palmitate (various concentrations) or vehicle into BD- or HFD-fed mice that had been fasted for 24 h. We selected 300 mM palmitate (3 μmol/g) for subsequent experiments, because it was the highest dose that elevated serum ALT levels in HFD-fed mice (Fig. S1B).

LPS (*E*. *coli* O55:B5; Sigma) was dissolved in PBS. We intraperitoneally injected 0.01 mL/g LPS (various concentrations) into BD-fed mice that had been fasted for 24 h. We analyzed the time course of serum ALT levels after 1.0 mg/kg LPS injection (Fig. S2B). At 24 h after LPS injection, serum ALT levels were the highest.

To investigate response against palmitate and LPS injection, we then intraperitoneally injected LPS (various concentrations) or 3 μmol/g palmitate (300 mM) 1 h after 3 μmol/g palmitate (300 mM) or LPS injection, respectively, into the mice. We collected the blood and tissue samples 24 h after the final injection. To establish an effective working LPS dose, various concentrations (0.01 mg/kg, 0.025 mg/kg, 0.05 mg/kg, 0.1 mg/kg and 1.0 mg/kg) of LPS were intraperitoneally injected into BD-fed mice (Fig. S2C). Though vehicle-0.025 mg/kg LPS injection did not change serum ALT levels compared with vehicle-PBS injection, palmitate-0.025 mg/kg LPS injection significantly up-regulated serum ALT levels compared with those following vehicle-0.025 mg/kg LPS or palmitate-PBS injection. Therefore, we used 0.025 mg/kg LPS for the experiments.

For analysis of long-term palmitate or LPS treatment, we intraperitoneally injected 3 μmol/g palmitate (300 mM), LPS (0.025 mg/kg), vehicle or PBS twice a day for 2 weeks into *ad libitum* BD- or HFD-fed mice. We collected blood and tissue samples 3 h after the final injection into *ad libitum*-fed mice.

#### Histologic and immunohistochemical evaluations

Formalin-fixed, paraffin-embedded sections (5 μm thick) were deparaffinized and dehydrated. The sections were then autoclaved in 10 mM citrate buffer (pH 6.0) and incubated with normal swine serum. The samples were exposed to primary antibodies anti- NE (1:1000, Abcam), anti-F4/80 (1:100, eBioscience), anti-αSMA (1:200, Abcam) and cleaved caspase 3 (1:100, Cell Signaling) overnight at 4 °C. This was followed sequentially by incubation with biotinylated secondary antibodies and avidin-biotinyl-peroxidase complex using the Vectastain ABC kit (Vector Laboratories). The peroxidase reaction was developed using diaminobenzidine tetrahydrochloride plus 0.02% hydrogen peroxide as the chromogen, and nuclear counterstaining was performed with hematoxylin. Sections stained with the secondary antibody alone served as the negative control. Apoptosis was assessed by TUNEL staining of paraffin-embedded slides.

Average numbers of NE-, cleaved caspase- and TUNEL-positive cells (×100) and F4/80-positive cells (×200) were examined in five fields per specimen. Average % of NE-, cleaved caspase- and TUNEL-positive cells (×100) and F4/80-positive cells (×200) were calculated as the average numbers of positive cells / hepatocytes in five fields per specimen. Average positive area for αSMA (×100) and sirius red (×200) were examined in five fields per specimen and quantified using Adobe Photoshop imaging software (Adobe Systems).

#### Western blotting

Livers were dissolved in M-PER Mammalian Protein Extraction Reagent (Thermo Fisher Scientific). After quantification by Bio-Rad protein assay (Bio-Rad), equal volumes of protein were electrophoresed by SDS-PAGE and electrophoretically transferred to Immobilon-P membranes (GE Healthcare BioSciences). The membranes were blocked with blocking reagent (TOYOBO) and incubated with anti-αSMA (1:400, Abcam) antibody in Can Get Signal Solution1® (TOYOBO). After the membranes were treated with the rabbit peroxidase-conjugated secondary antibody, specific signals were detected using an ECL detection kit (GE Healthcare BioSciences). Expression of GAPDH was used as the internal control.

#### Quantitative real-time PCR

Total RNA was extracted from liver tissue samples using the RNeasy Mini Kit and from cultured cells using QIAzol Lysis Reagent (Qiagen) and the RNeasy Mini Kit (Qiagen), according to the manufacturer’s instructions. cDNA was generated using high-capacity cDNA Reverse Transcriptase (Applied Biosystems). mRNA expression of Ccl2, Ccl3, Cxcl1, Cxcl2, Cxcl10, collagen 1a1, DR5, IL-1β, tissue inhibitor of metalloproteinases-1, TGFβ1, TNFα, αSMA (encoded by Acta2) and β-actin was assessed by quantitative real-time PCR (7500 Fast real-time PCR System, Applied Biosystems) with appropriate TaqMan probes (Sigma-Aldrich). The quantities of the amplified products were normalized to the quantity of β-actin.

#### Endotoxin assay

Plasma endotoxin levels were measured. Briefly, samples were heated at 75 °C for 5 min, and endotoxin levels were determined using a commercially available limulus amebocyte lysate chromogenic endpoint assay with a concentration range of 0.04–10 EU/ml (Hycult Biotech).

### Studies of human

#### Human subjects

For histopathologic analysis, fasted human blood was collected from 498 patients with liver biopsy-diagnosed NAFLD. The patients were evaluated at two medical centers Hiroshima University Hospital and Nara City Hospital, Japan, and enrolled between August 2003 and September 2014. For the endotoxin activity assay (EAA) study, fasted human blood was collected from 127 patients with liver biopsy-diagnosed NAFLD (fibrosis stages 0, 1 and 2). The patients were evaluated at Yokohama City University Hospital, Japan, and enrolled between January 2014 and July 2017.

Human subjects were patients with liver biopsy-diagnosed NAFLD. Subjects with a history of excessive alcohol consumption (weekly consumption 140 g for men or 70 g for women), other liver diseases, such as chronic hepatitis, drug use associated with fatty liver, weight reduction or thyroid disorders were excluded. All recruitment and experimental procedures adhered to the Declaration of Helsinki. The experimental protocol was approved by the institutional review boards of Yokohama City University Hospital, Hiroshima University Hospital, Nara City Hospital, Japan; prior to participating, all patients provided their informed consent.

#### Histologic examination and liver histology

Liver biopsy samples were obtained from all patients with NAFLD using a 16- or 18-G needle biopsy kit according to a standard protocol. Two specimens were obtained from each patient to acquire a sample of sufficient size for analysis and to reduce histological errors. An adequate liver biopsy sample was defined as >16 mm in length and/or with >10 portal tracts. Livers were assessed histologically by two pathologists. Macrovesicular steatosis affecting at least 5% of hepatocytes was observed in all patients with NAFLD. Patients with steatosis, inflammation, ballooned hepatocytes and pericellular/perisinusoidal fibrosis were classified as having NASH^43^. For the histological feature scoring system, we used the NAFLD activity score^44^. Steatosis affecting <5%, 5%–33%, 33%–66% and >66% of hepatocytes was classified as grades 0, 1, 2 and 3, respectively. Lobular inflammation was graded according to the number of inflammatory foci per field of view at a magnification of ×200, with 0, <2, 2–4 and >4 foci per field classified as grades 0, 1, 2 and 3, respectively. Hepatocellular ballooning involving no, few and many cells was classified as grades 0, 1 and 2, respectively. Fibrosis severity was assessed as previously described^45^.

#### Endotoxin activity assay (EAA)

Endotoxin activity (EA) in whole blood was measured as described elsewhere^46–48^ by use of a murine IgM monoclonal antibody raised against lipid A of *Escherichia coli* J5. Whole blood samples (40 μL) were incubated in duplicate with saturating concentrations of antibody and then stimulated with zymosan. The resulting respiratory burst activity was detected as light release from the lumiphor luminol by use of a chemiluminometer (TORAY). The LPS/anti-LPS complex primes the patient’s neutrophils for an augmented response to stimulation with zymosan. By measuring basal (no antibody) and maximally stimulated responses in the same blood sample, the EA of the test specimen can be calculated by integrating the chemiluminescence over time. Levels are expressed as EA units and represent the mean of duplicate determinations from the same sample. EA is expressed in relative units (0–1.0) derived from the integral of the basal and stimulated chemiluminescent response.

### Statistical analysis

Data are presented as means ± standard deviation (SD). Analyses between the two groups were performed by unpaired two-tailed Student’s t test. Data sets involving more than two groups were assessed by ANOVA with Scheffe’s multiple testing correction. Correlation analysis was performed by Spearman’s rank correlation coefficient. Values of P < 0.05 were considered significant. All statistical analyses were performed using JMP v11.2.0 (SAS Institute).

### Other methods

Please see the Supplementary Materials and Methods for a detailed description of other experimental procedures.

## Electronic supplementary material

Supplementary Information
