# Supplementary material for: Palmitate-induced lipotoxicity is crucial for the pathogenesis of nonalcoholic fatty liver disease in cooperation with gut-derived endotoxin
Source: Sci Rep. 2018 Jul 27;8:11365. doi: 10.1038/s41598-018-29735-6 (PMC6063851; doi:10.1038/s41598-018-29735-6)
Supplement: Supplementary file 1 — Supplementary Information [file 41598_2018_29735_MOESM1_ESM.docx]

**Supplementary Information**

**Palmitate-induced lipotoxicity is crucial for the pathogenesis of nonalcoholic fatty liver disease in cooperation with gut-derived endotoxin.**

Yuji Ogawa, Kento Imajo, Yasushi Honda, Takaomi Kessoku, Wataru Tomeno, Shingo Kato, Koji Fujita, Masato Yoneda, Satoru Saito, Yusuke Saigusa, Hideyuki Hyogo, Yoshio Sumida, Yoshito Itoh, Kosei Eguchi, Takeharu Yamanaka, Koichiro Wada, Atsushi Nakajima

**SUPPLEMENTARY METHODS**

**Studies of mouse**

*Gut sterilization*

Mice were treated with ampicillin (1 g/L; Sigma), neomycin (1 g/L; Sigma), metronidazole (1 g/L; Sigma) and vancomycin (500 mg/L; Sigma) in drinking water for 4 weeks. This was followed by palmitate injection.

*Clodronate liposomes*

To deplete circulating monocytes, mice were injected in the tail vein with clodronate liposomes (FormuMax Scientific; 0.2 mL/body for BD-fed mice and 0.4 mL/body for HFD-fed mice). We used empty control liposomes (FormuMax Scientific) as a negative control. Either clodronate or control liposomes were administered 48 h prior to palmitate or vehicle injection.

*Ly-6G*

To decrease circulating neutrophils, mice were intraperitoneally injected with Ly-6G (Bio X cell; 0.2 mL/body for BD-fed mice and 0.4 mL/body for HFD-fed mice). We used IgG2a isotype control (Bio X cell) as a negative control. Either Ly-6G or control IgG was administered 24 h prior to palmitate or vehicle injection.

*Histological scoring*

Nonalcoholic fatty liver disease activity score (NAS) and fibrosis stage were scored as previously described ^1, 2^.

*Biochemistry*

Serum FFA levels were measured by the acyl-CoA synthetase-acyl-CoA oxidase method (Wako Pure Chemical Industries) according to the manufacturer’s instructions. Serum ALT levels were measured by a local laboratory for clinical examinations (SRL). For assessment of insulin resistance, homeostasis model assessment of insulin resistance was calculated as fasting insulin level (µU/mL) × fasting blood glucose level (mg/dL) / 405.

*Triglyceride (TG) determination in the liver*

Lipids were extracted from liver tissue (50 mg) using a chloroform (C):methanol (M) mixture (C:M = 2:1) as described by Folch et al. ^3^. TG levels in liver were measured using an L Type Wako TG kit (Wako).

*Microarray*

Total RNA was isolated using a standard RNA extraction protocol. Microarray analysis was performed using Mouse Gene 2.0 ST Array (Affymetrix) according to the manufacturer’s instructions. Briefly, first and second strand cDNA syntheses were performed using WT PLUS Reagent Kit (Affymetrix). cRNA was generated using WT PLUS Reagent Kit followed by cRNA cleanup using magnetic beads (Affymetrix). cRNA was confirmed by Bioanalyzer (Agilent). After second cycle ss-cDNA synthesis using cRNA, ss-cDNA was labeled, fragmented and hybridized to a gene chip array. After hybridization to the gene chip, data from a genechip Scanner 3000 7G (Affymetrix) were analyzed using Expression ConsoleTM Software ver.1.3.0 (Affymetrix). Analysis was performed as paired analysis comparing palmitate- and vehicle-treated mice.

*In situ hybridization*

The excised liver tissues from palmitate- and vehicle-treated mice were fixed in 10% paraformaldehyde phosphate buffer solution for 48 h and then processed for paraffin embedding. The paraffin blocks were cut into 3-µm-thick sections and were mounted on silane-coated glass slides. After deparaffinization, the sections were dipped into boiling pretreat solution for 10 min. After washing in distilled water, protease solution was added to the sections for 20 min at 40°C. After washing with PBS twice, the sections were hybridized with QuantiGene ViewRNA Type 1 mouse Cxcl-2 probe (Affymetrix) or mouse Ccl-2 probe (Affymetrix) for 2 h at 40°C. Signal amplification and signal detection with Fast Red substrate were performed according to manufacturer’s instructions (QuantiGene ViewRNA ISH Tissue Assay, Affymetrix).

Endotoxin assay

Plasma endotoxin levels were evaluated using limulus amebocyte lysate chromogenic endpoint assay with a concentration range of 0.04–10 EU/mL (Hycult Biotech). Then, EU levels obtained were converted to pg/mL with 1EU = 100 pg/m ^4^.

**Studies of human**

*Biochemistry*

After a 12-h fast, venous blood samples were collected. Plasma glucose and serum ALT, aspartate aminotransferase, total cholesterol, low-density lipoprotein cholesterol, high-density lipoprotein cholesterol and TG levels were measured using standard techniques. Serum FFAs were measured with the acyl-CoA synthetase-acyl-CoA oxidase method. Serum type IV collagen 7S was measured using radioimmunoassay kit (Sceti Medical labo). Serum hyaluronic acid was measured using latex agglutination immunoassay (Mitsubishi Chemical).

Endotoxin

Endotoxin activity in whole blood was measured using endotoxin activity assay (EAA). Plasma endotoxin levels of 10 in the “FFA-high” patient with NAFLD were measured in two ways. First, plasma endotoxin levels were evaluated using turbidimetric time assay, endotoxin single test (Wako). Second, plasma endotoxin levels were evaluated using limulus amebocyte lysate chromogenic endpoint assay with a concentration range of 0.04–10 EU/mL (Hycult Biotech).

**Supplementary Figure S1**

**
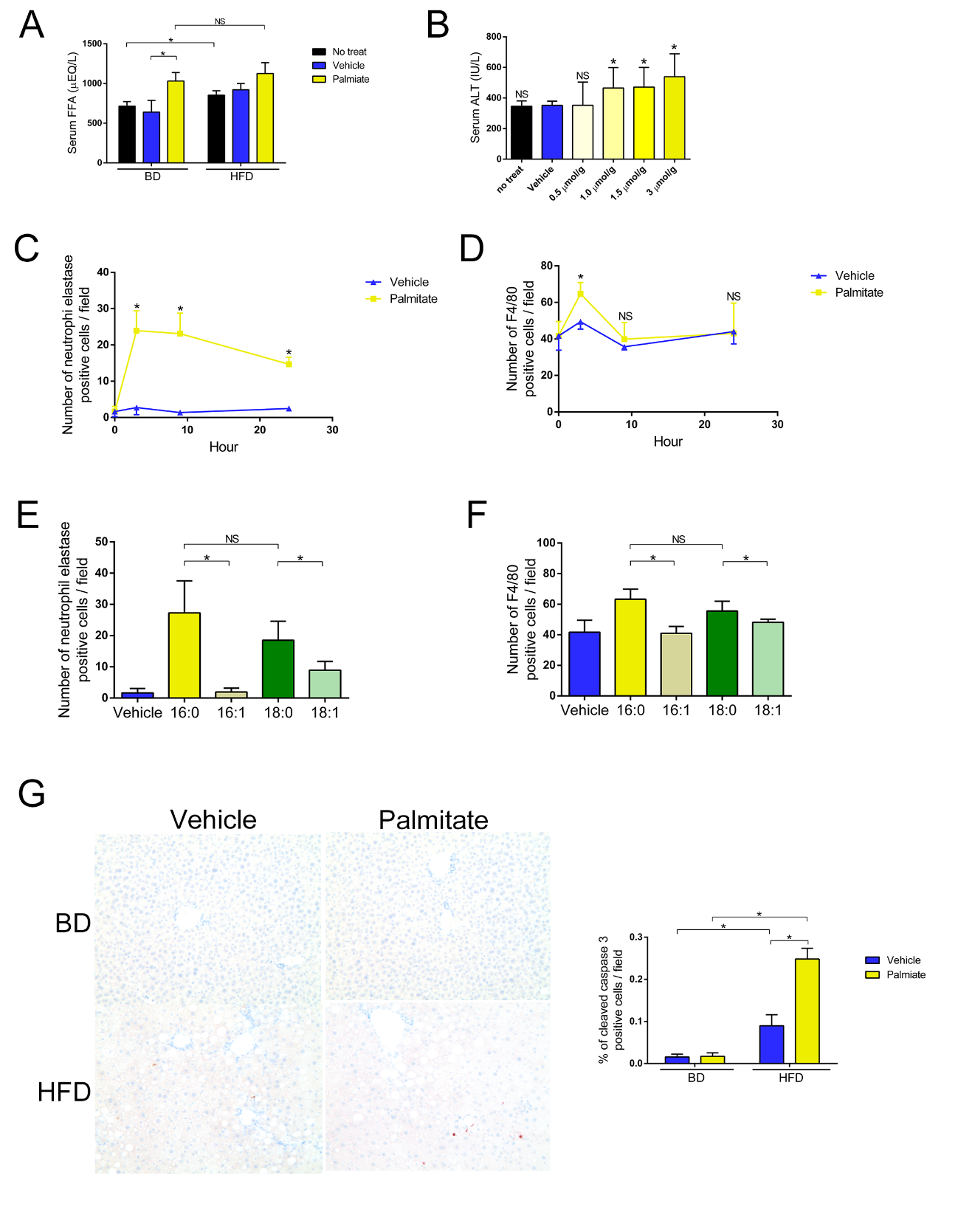
**

**Figure S1. Related to Figure 1. Palmitate induced inflammation in the liver.**

(A) Serum FFA levels were measured 3 h after 3 μmol/g palmitate (300mM) or vehicle injection or no treatment in BD- and HFD-fed 28-week-old WT mice.

(B) Serum ALT levels were measured 24 h after treatment with various concentrations of palmitate (0.5 μmol/g [50 mM palmitate], 1.0 μmol/g [100 mM palmitate], 1.5 μmol/g [150 mM palmitate], 3 μmol/g [300 mM palmitate]) or vehicle injection or no treatment in HFD-fed 28-week-old WT mice.

(C, D) Immunohistochemical detection of average numbers of NE- (×100) and F4/80-positive (×200) cells 0, 3, 9 and 24 h after 3 μmol/g palmitate or vehicle injection in 28-week-old BD-fed WT mice.

(E, F) Immunohistochemical detection of average numbers of NE- (×100) and F4/80-positive (×200) cells 3 h after a single intraperitoneal injection of 3 μmol/g various fatty acids (palmitate [C16:0], stearate [C18:0], palmitoleate [C8:1] and oleate [C18:1]) or vehicle in BD-fed 28-week-old WT mice.

(G) Immunohistochemical detection of cleaved caspase 3 (×200) in representative liver samples and average % of cleaved caspase 3-positive cells (×100) 24 h after palmitate or vehicle injection were examined in BD-fed 28-week-old WT mice. Scale bars, 100 μm.

n=5 mice per group.

Data represent the mean ± SD. *P < 0.05. NS, not significant.

**Supplementary Figure S2**

**
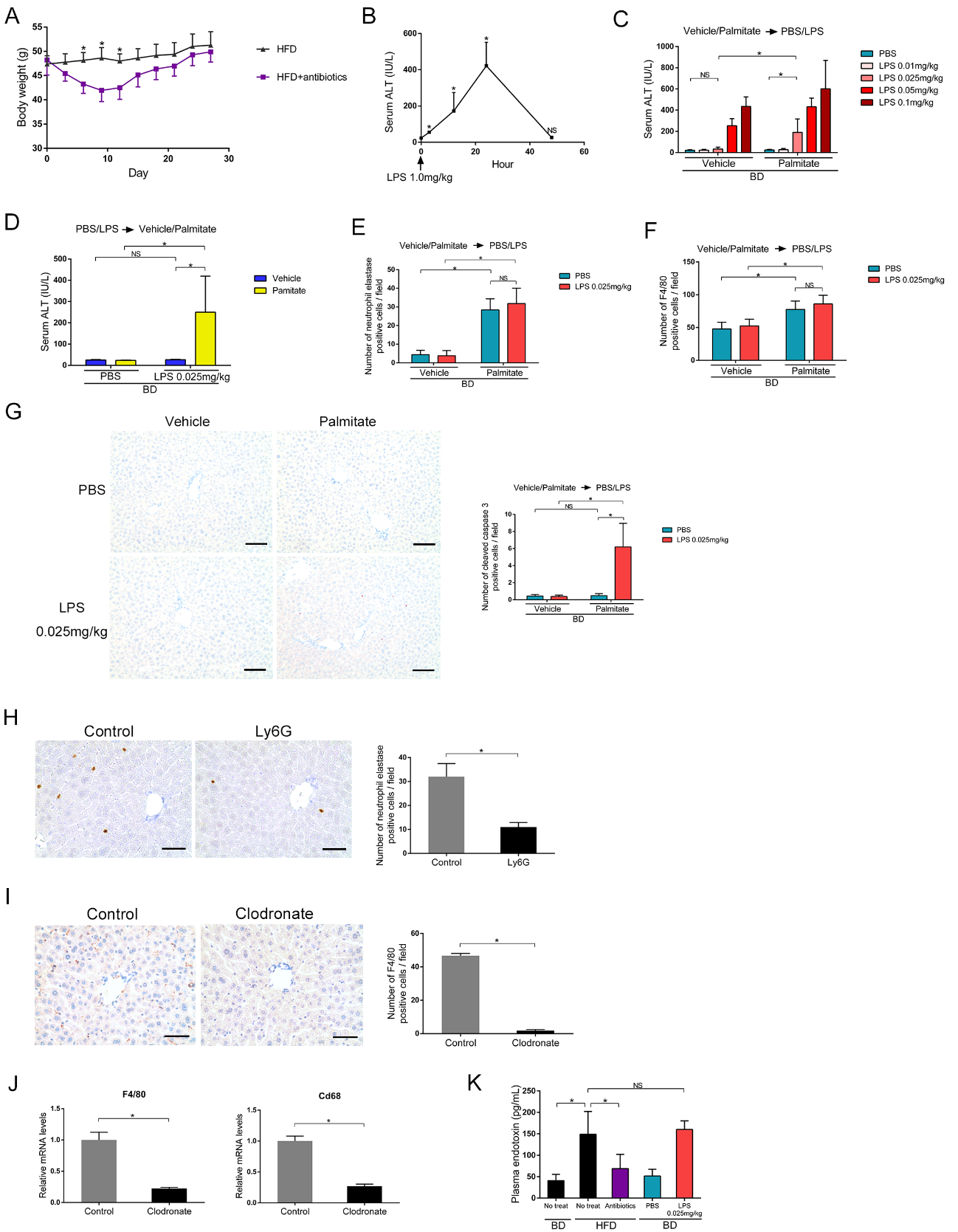
**

**Figure S2. Related to Figure 2. Palmitate plus LPS injection elevated serum ALT levels.**

(A) Growth curves of HFD-fed WT mice that were gut sterilized or untreated between 24 and 28 weeks of age.

(B) Serum ALT levels were measured 0, 3, 9 and 24 h after LPS 1.0 mg/kg injection into 28-week-old BD-fed mice.

(C) Serum ALT levels were measured 24 h after various concentrations of LPS (0.01, 0.025, 0.05 and 0.1 mg/kg) or PBS. LPS or PBS was intraperitoneally injected into 28-week-old BD-fed mice 1 h after intraperitoneal palmitate or vehicle injection.

(D) Serum ALT levels were measured 24 h after palmitate injection. Palmitate was injected 1 h after 0.025 mg/kg LPS or PBS injection in 28-week-old BD-fed mice.

(E, F) Immunohistochemical detection of average numbers of NE- (×100) and F4/80-positive (×200) cells 3 h after 0.025 mg/kg LPS or PBS injection. LPS or PBS was injected 1 h after vehicle or palmitate injection in 28-week-old BD-fed mice.

(G) Representative immunostaining (×200) and immunohistochemical detection of average numbers of cleaved caspase 3-positive cells (×100) 24 h after palmitate/vehicle and 0.025 mg/kg LPS/PBS injection in 28-week-old BD-fed mice. LPS or PBS was injected 1 h after vehicle or palmitate injection. Scale bars, 100 μm.

(H) Ly-6G or control IgG was intraperitoneally injected 24 h prior to palmitate injection in BD-fed mice. Representative immunostaining (×400) and immunohistochemical detection of average numbers of NE-positive cells (×100) in the liver 3 h after palmitate injection in 28-week-old BD-fed WT mice. Scale bars, 50 μm.

(I) Clodronate or control liposomes were injected into the tail vein. Representative immunostaining (×400) and immunohistochemical detection of average numbers of F4/80-positive cells (×200) in the liver 48 h after clodronate or control liposome injection in 28-week-old BD-fed WT mice. Scale bars, 50 μm.

(J) mRNA levels of macrophage markers (F4/80 and Cd68) in whole liver 48 h after clodronate or control liposome injection in 28-week-old BD-fed WT mice.

(K) Plasma endotoxin levels were measured in 28-week-old BD-fed WT mice 24 h after a single intraperitoneal injection of 0.025 mg/kg LPS or PBS or no treatment and in HFD-fed 28-week-old WT mice 4 weeks after gut-sterilization or no treatment. Plasma endotoxin levels “EU/mL” obtained were converted to “pg/mL”.

n=5 mice per group.

Data represent the mean ± SD. *P < 0.05. NS, not significant.

**Supplementary Figure S3**

**
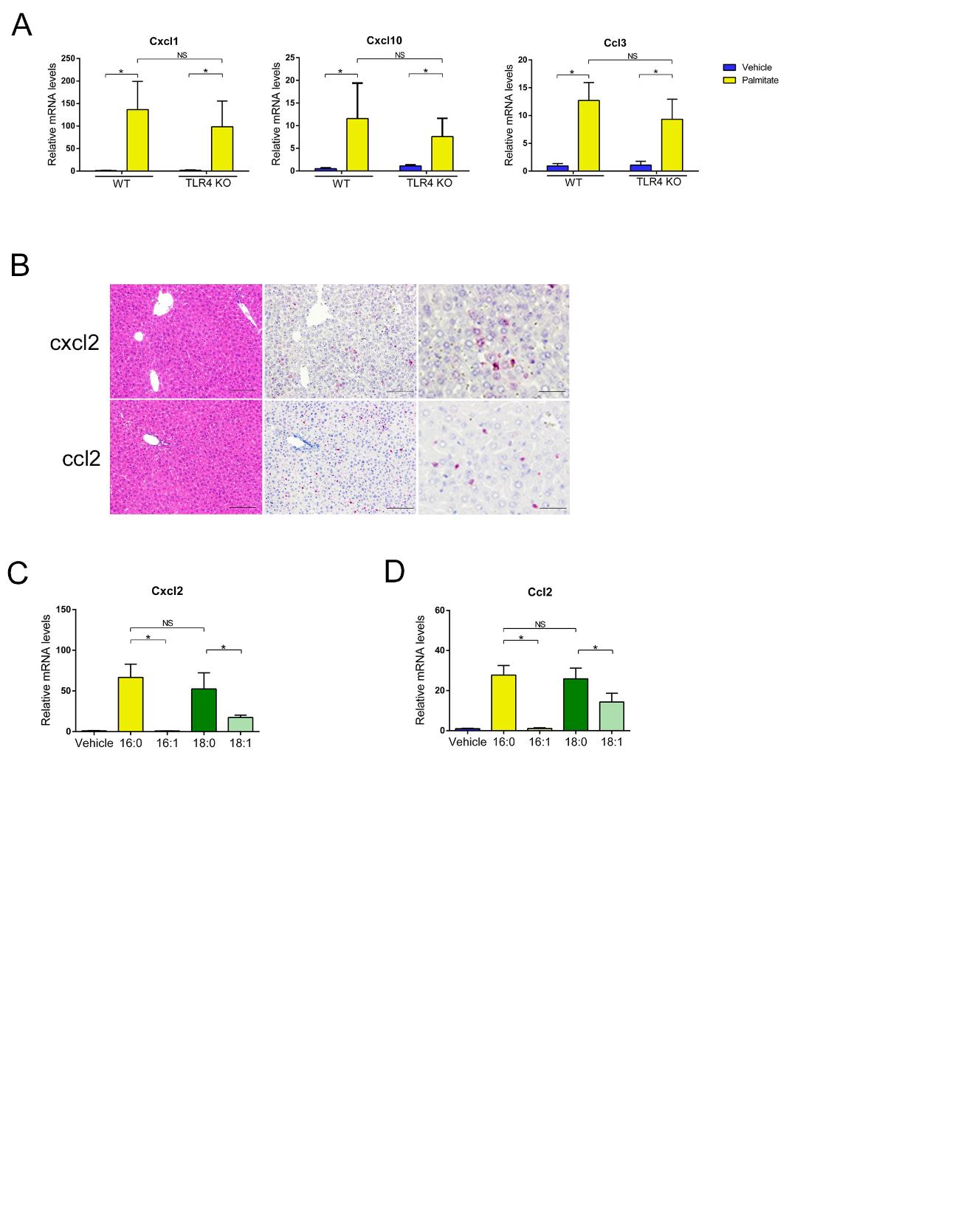
**

**Figure S3. Related to Figure 3. Palmitate induced chemokines in the liver via the TLR4 pathway.**

(A) Cxcl1, Cxcl10 and Ccl3 mRNA expression levels in whole liver were analyzed by qPCR 3 h after palmitate or vehicle injection in 8-week-old BD-fed WT and TLR4 KO mice.

(B) *In situ* hybridization for the localization of Cxcl2 and Ccl2 mRNA in whole liver 3 h after palmitate or vehicle injection in 28-week-old BD-fed WT mice was performed. Middle (×200, scale bars, 100 μm), right (×400, scale bars, 50 μm). (Left) Hematoxylin-eosin, (×200, scale bars, 100 μm).

(C, D) Cxcl2 and Ccl2 mRNA expression levels in whole liver were analyzed by qPCR 3 h after injection with various fatty acids (palmitate [C16:0], stearate [C18:0], palmitoleate [C8:1] and oleate [C18:1]) or vehicle in 28-week-old BD-fed WT mice.

n=5 mice per group.

Data represent the mean ± SD. *P < 0.05. NS, not significant.

**Supplementary Figure S4**

**
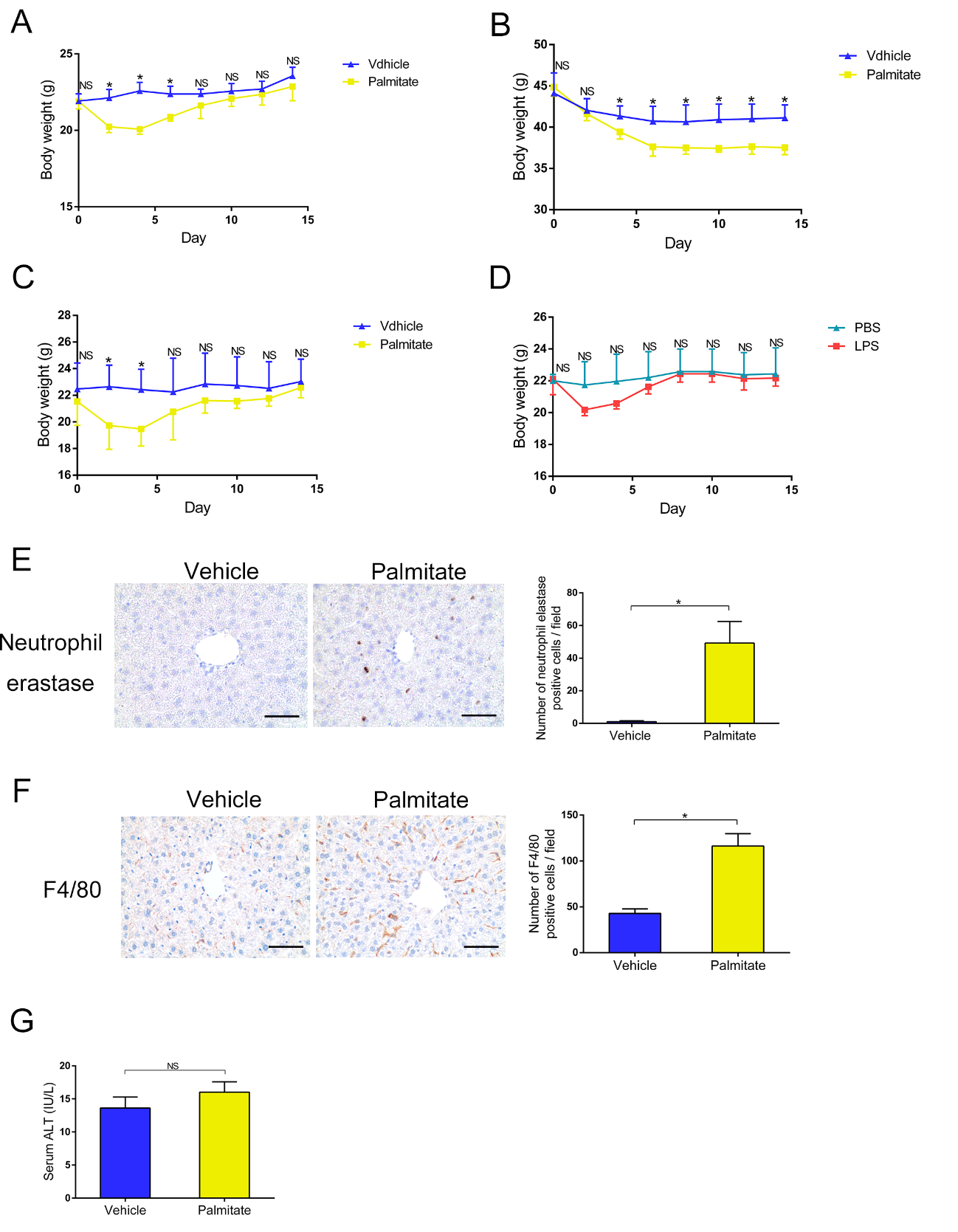
**

**Figure S4. Related to Figure 4. Long-term palmitate or LPS injection.**

For the analysis of long-term injection, we intraperitoneally injected 3 μmol/g palmitate (300mM), vehicle, 0.025 mg/kg LPS or PBS twice a day for 2 weeks into *ad libitum*-fed mice.

(A) Growth curves of long-term (2 weeks) palmitate- or vehicle-injected 10-week-old BD-fed WT mice.

(B) Growth curves of long-term (2 weeks) palmitate- or vehicle-injected 30-week-old HFD-fed WT mice.

(C) Growth curves of long-term (2 weeks) palmitate- or vehicle-injected 10-week-old BD-fed TLR4 KO mice.

(D) Growth curves of long-term (2 weeks) 0.025 mg/kg LPS- or PBS-injected 10-week-old BD-fed WT mice.

(E, F) Representative immunostaining of NE and F4/80 (×400) in the liver and average numbers of NE- (×100) and F4/80-positive (×200) cells in long term (2 weeks) palmitate- or vehicle-injected 10-week-old BD-fed WT mice. Scale bars, 50 μm.

(G) Serum ALT levels were measured in long-term (2 weeks) palmitate- or vehicle-injected 10-week-old BD-fed WT mice.

n=5 mice per group.

Data represent the mean ± SD. *P < 0.05. NS, not significant.

**
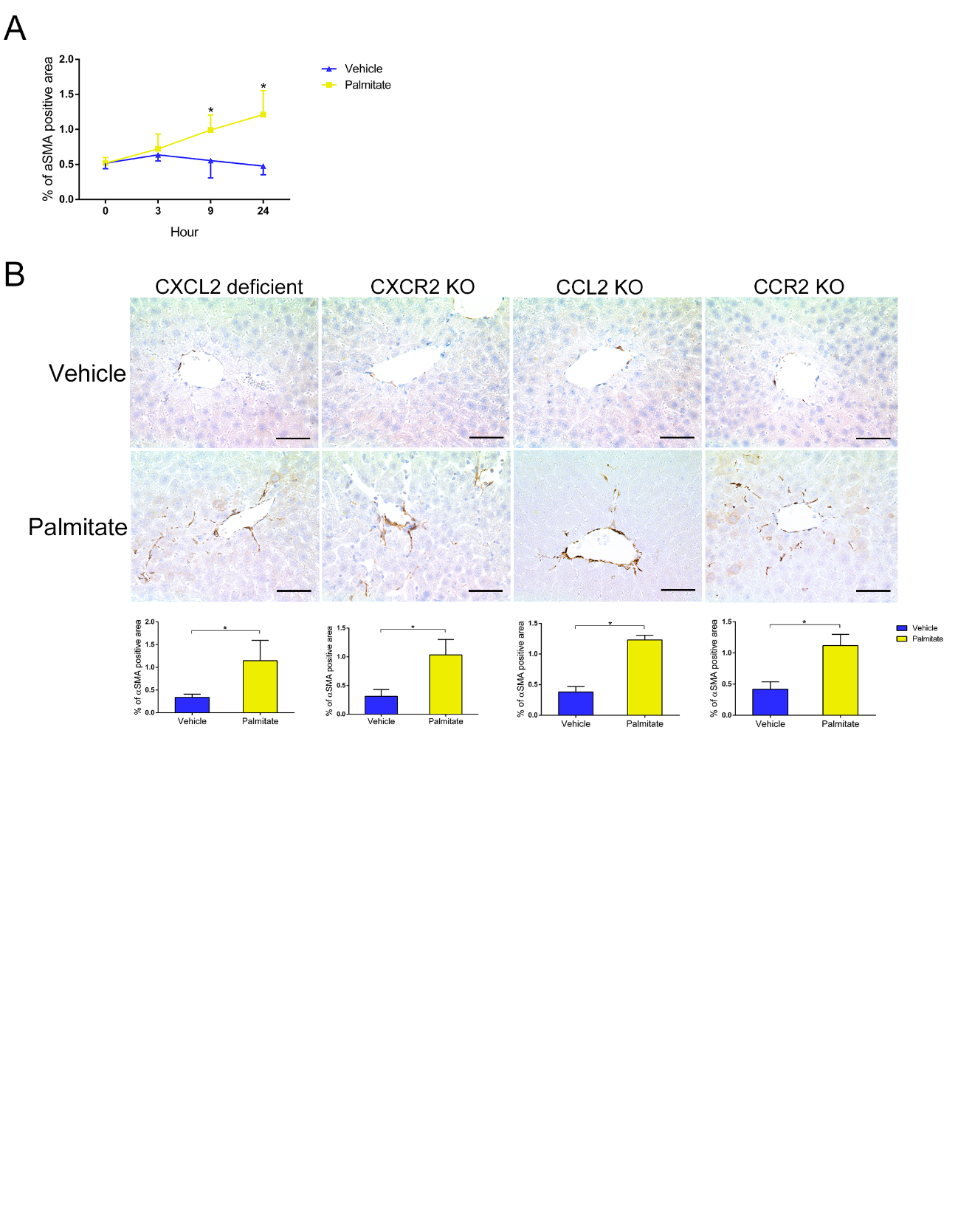
Supplementary Figure S5**

**Figure S5. Related to Figure 4. A single injection of palmitate.**

(A) αSMA-positive area (×100) was examined 0, 3, 9 and 24 h after a single palmitate or vehicle injection in 8-week-old BD-fed WT mice.

(B) Representative immunostaining (×400) and immunohistochemical detection of average αSMA positive area (×100) in the liver 24 h after a single palmitate or vehicle injection in 8-week-old BD-fed CXCL2 deficient, CXCR2 KO, CCL2 KO and CCR2 KO mice. Scale bars, 50 μm.

n=5 mice per group.

Data represent the mean ± SD. *P < 0.05. NS, not significant.

|  | BD (8W) | BD (28W) | HFD (28W) | HFD (28W)  +Abs |
| --- | --- | --- | --- | --- |
| Body weight (g) | 20.4±1.1 | 28.0±0.5* | 43.4±1.2*^#^ | 43.8±3.4*^#^ |
| Liver weight (g) | 0.97±0.06 | 1.14±0.09* | 2.86±0.10*^#^ | 2.77±0.53*^#^ |
| Hepatic TGs (mg/g tissue) | 39.3±3.6 | 40.0±1.1 | 170.3±6.7*^#^ | 170.6±7.1*^#^ |
| Visceral fat (g) | 0.20±0.05 | 0.29±0.03* | 1.38±0.09*^#^ | 1.43±0.18*^#^ |
| Subcutaneous fat (g) | 0.15±0.03 | 0.23±0.03* | 1.26±0.07*^#^ | 1.31±0.05*^#^ |
| HOMA-IR | 0.77±0.15 | 0.82±0.30 | 14.2±2.4*^#^ | 11.8±5.1*^#^ |

**Table S1.** **Related to Figure 1, 2 and 4.** Characteristics of BD- and HFD-fed WT mice.

Results are presented as means ± SD.

*P < 0.05 compared with BD (8W).

^#^P < 0.05 compared with BD (28W).

Abs, antibiotics; BD, basal diet; HFD, high-fat diet; HOMA-IR, homeostasis model assessment for insulin resistance; TGs, triglycerides; WT, wild-type.

| Probe set ID | Gene symbol | Fold change (range) |
| --- | --- | --- |
| Chemokine signaling pathway | | |
| 17438987 | Cxcl1 | 3.919 (42.97 to 650.3) |
| 17449718 | Cxcl10 | 2.926 (23.08 to 175.4) |
| 17254041 | Ccl2 | 2.637 (142.8 to 22.99) |
| 17266967 | Ccl3 | 2.407 (11.77 to 62.47) |
| 17438995 | Cxcl2 | 0.910 (15.99 to 30.05) |
| Innate immune response | | |
| 17414836 | Tlr4 | 0.489 (18.79 to 26.39) |
| 17353747 | Cd14 | 0.897 (14.72 to 27.42) |
| Leukocyte transendothelial migration | | |
| 17515074 | Icam1 | 2.142 (54.17 to 239.1) |
| 17409649 | Vcam1 | −0.027 (118.3 to 116.1) |
| Cytokines | | |
| 17391554 | Il1a | 1.699 (37.12 to 120.5) |
| 17344309 | Tnf | 0.978 (16.99 to 33.47) |
| 17391565 | Il1b | 0.958 (28.93 to 56.23) |
| 17301697 | Tnfrs10b | 0.312 (11.51 to 14.29) |
| 17415361 | Ifna11 | −1.600 (7.074 to 2.332) |
| Apoptosis | | |
| 17281219 | Nfkbia | 1.207 (338.5 to 781.6) |
| 17501041 | Casp3 | 0.635 (43.39 to 67.42) |
| 17301697 | Tnfrsf10b | 0.312 (11.51 to 14.29) |
| Liver fibrosis | | |
| 17533713 | Timp1 | 0.697 (7.926 to 12.85) |
| 17364098 | Acta2 | 0.374 (58.60 to 75.94) |
| 1747534 | Tgfb1 | 0.246 (99.40 to 117.9) |

**Table S2. Related to Figure 2.** Three different whole livers were used for microarray analysis 3 h after palmitate or vehicle treatment.

|  | BD | | | HFD | | |
| --- | --- | --- | --- | --- | --- | --- |
| Histological scoring | No teat | single  palmitate injection | long-term palmitate injection | No teat | single  palmitate injection | long-term palmitate injection |
| Steatosis | 0±0 | 0±0 | 0.4±0.5 | 1.4±0.5 | 1.6±0.5 | 1.8±0.8 |
| Lobular inflammation | 0±0 | 0.8±0.4* | 1.4±0.5* | 0.2±0.4 | 1.2±0.4^#^ | 1.6±0.5^#^ |
| Hepatocyte Ballooning | 0±0 | 0±0 | 0.2±0.4 | 0±0 | 0±0 | 0.2±0.4 |
| NAS | 0±0 | 0.8±0.4* | 2.0±1.2* | 1.6±0.8 | 2.8±0.8 | 3.6±0.8^#^ |
| Fibrosis stage | 0±0 | 0±0 | 0.8±0.4* | 0±0 | 0±0 | 1.2±0.4^#^ |

**Table S3.** **Related to Figure 1, 2 and 4.** Histological scoring of BD- and HFD-fed WT mice. NAS, nonalcoholic fatty liver disease activity score.

Data are the mean ± SD (n = 5 each group).

* p<0.05 vs BD no treat mice

^#^ p<0.05 vs HFD no treat mice

|  | EAA-low/FFA-low  (n=45) | EAA-high/FFA-low  (n=36) | EAA-low/FFA-high  (n=25) | EAA-high/FFA-high  (n=21) |
| --- | --- | --- | --- | --- |
| Age (years) | 55.6±14.7 | 53.6±13.8 | 52.8±13.4 | 54.4±16.1 |
| Sex (male/female) | 26/19 | 24/12 | 10/15 | 8/13 |
| BMI (kg/m^2^) | 28.3±4.10 | 28.0±4.04 | 28.0±4.54 | 28.8±3.81 |
| Platelet count (10^4^/μL) | 23.2±6.34 | 21.5±5.98 | 22.0±6.24 | 20.1±5.92 |
| FFA (μEq/L) | 549.7±148.1 | 563.6±147.2 | 1035.2±333.86 | 1215.7±514.88 |
| EAA | 0.104±0.042 | 0.266±0.096 | 0.041±0.008 | 0.282±0.076 |
| Diabetes (%) | 64.4 | 58.3 | 52.0 | 76.1 |
| Dyslipidemia (%) | 77.7 | 86.1 | 76.0 | 76.1 |
| Hypertension (%) | 60.0 | 41.6 | 52.0 | 42.8 |
| Steatosis |  |  |  |  |
| 0 | 0 | 0 | 0 | 0 |
| 1 | 19 | 16 | 12 | 6 |
| 2 | 22 | 12 | 8 | 12 |
| 3 | 4 | 8 | 5 | 3 |
| Lobular inflammation |  |  |  |  |
| 0 | 2 | 1 | 1 | 1 |
| 1 | 30 | 28 | 18 | 12 |
| 2 | 12 | 7 | 6 | 7 |
| 3 | 1 | 0 | 0 | 1 |
| Hepatocellular ballooning |  |  |  |  |
| 0 | 5 | 4 | 3 | 1 |
| 1 | 38 | 29 | 19 | 18 |
| 2 | 2 | 3 | 3 | 2 |
| Fibrosis |  |  |  |  |
| 0 | 3 | 1 | 1 | 0 |
| 1 | 21 | 21 | 14 | 11 |
| 2 | 21 | 14 | 10 | 10 |
| 3 | 0 | 0 | 0 | 0 |
| 4 | 0 | 0 | 0 | 0 |

**Table S4. Related to Figure 5.** Clinical and biochemical characteristics of patients with NAFLD.

Results are presented as means ± SD.

BMI, body mass index; EAA, endotoxin activity assay; FFA, free fatty acid; NAFLD, nonalcoholic fatty liver disease.

|  | “FFA-high and EAA-high” group | Other groups | P value |
| --- | --- | --- | --- |
| ALT (IU/L) | 84.4 ± 31.8 | 55.6 ± 29.1 | <0.001 |

**Table S5.** **Related to Figure 5.** Student’s t test between ”FFA-high and EAA-high” group and other groups (“FFA-low and EAA-low”, ”FFA-low and EAA-high”, and “FFA-high and EAA-low” group) for the serum ALT levels.

ALT, alanine aminotransferase; EAA, endotoxin activity assay; FFA, free fatty acid.

| Factor | β value | 95% CI | P value |
| --- | --- | --- | --- |
| Age | -0.91 | -1.27 ~ -0.55 | <0.001 |
| Body mass index | 1.31 | 0.09 ~ 2.53 | 0.03 |
| Platelet count (×10^4^/μL) | -0.73 | -1.55 ~ 0.08 | 0.07 |
| FFA (μEQ/L) | -0.02 | -0.054 ~ 0.009 | 0.16 |
| EAA | -72.7 | -173.8 ~ 28.3 | 0.15 |
| FFA (μEQ/L) x EAA | 0.15 | 0.02 ~ 0.28 | 0.02 |

**Table S6.** **Related to Figure 5.** Multiple linear regression analysis for serum ALT levels. ALT, alanine aminotransferase; CI, confidence interval; EAA, endotoxin activity assay; FFA, free fatty acid.

|  | EAA | Limulus amebocyte lysate chromogenic endpoint assay  (EU/mL) | Turbidimetric time assay  (pg/mL) |
| --- | --- | --- | --- |
| Endotoxin | 0.31 ± 0.04 | 1.85 ± 1.30 | <0.8 ± 0.0 |

**Table S7.** **Related to Figure 5.** Circulating endotoxin levels of the “FFA-high” patients with NAFLD. Patients of each group are the same patients (n=10). EAA, endotoxin activity assay; FFA, free fatty acid; NAFLD: nonalcoholic fatty liver disease

|  | NAFLD (n=498) |
| --- | --- |
| Age (years) | 51.8±14.1 |
| Sex (male/female) | 282/216 |
| BMI (kg/m^2^) | 28.3±4.7 |
| Platelet count (/10^4^ μL) | 23.2±17.8 |
| AST (IU/L) | 54.2±33.4 |
| ALT (IU/L) | 87.4±56.8 |
| T-Chol (mg/dL) | 212.8±42.5 |
| Triglycerides (mg/dL) | 175.0±114.7 |
| HDL (mg/dL) | 52.6±14.6 |
| FFAs (μEq/L) | 645.2±238.1 |
| Type 4 collagen 7S (ng/mL) | 4.5±2.6 |
| Hyaluronic acid (ng/mL) | 60.7±187.9 |
| Ferritin (ng/mL) | 232.8±230.6 |
| Fasting blood glucose (mg/dL) | 118.7±40.6 |
| Fasting insulin (mU/mL) | 16.2±12.9 |
| Diabetes (%) | 76.2 |
| Dyslipidemia (%) | 81.1 |
| Hypertension (%) | 57.7 |
| Steatosis |  |
| 0 | 0 |
| 1 | 273 |
| 2 | 133 |
| 3 | 92 |
| Lobular inflammation |  |
| 0 | 8 |
| 1 | 299 |
| 2 | 162 |
| 3 | 29 |
| Hepatocellular ballooning |  |
| 0 | 15 |
| 1 | 290 |
| 2 | 193 |
| Fibrosis |  |
| 0 | 8 |
| 1 | 107 |
| 2 | 226 |
| 3 | 143 |
| 4 | 14 |

**Table S8.** **Related to Table 1.** Clinical and biochemical characteristics of patients with NAFLD.

Results are presented as means ± SD.

AST, aspartate aminotransferase; ALT, alanine aminotransferase; BMI, body mass index; FFA, free fatty acid; HDL, high-density lipoprotein cholesterol; NAFLD, nonalcoholic fatty liver disease; T-Chol, total cholesterol.

| Factor | β value | 95% CI | P value |
| --- | --- | --- | --- |
| Body mass index | 0.015 | 0.001 ~ 0.030 | 0.03 |
| Platelet count | -0.007 | -0.011 ~ -0.003 | <0.001 |
| AST | 0.005 | 0.002 ~ 0.007 | <0.001 |
| Triglyceride | -0.0008 | -0.0014 ~ -0.0002 | 0.005 |
| FFA | 0.0005 | 0.0002 ~ 0.0008 | <0.001 |
| Type IV collagen 7S | 0.04 | 0.01 ~ 0.06 | 0.002 |

**Table S9.** **Related to Table 1.** Multiple linear regression analysis for liver fibrosis stage. AST, aspartate aminotransferase; CI, confidence interval; FFA, free fatty acid.

**REFERENCES**

1 Kleiner, D. E. et al. Design and validation of a histological scoring system for nonalcoholic fatty liver disease. Hepatology 41, 1313-1321, doi:10.1002/hep.20701 (2005).

2 Brunt, E. M. Nonalcoholic steatohepatitis: definition and pathology. Seminars in liver disease 21, 3-16 (2001).

3 Folch, J., Lees, M. & Sloane Stanley, G. H. A simple method for the isolation and purification of total lipides from animal tissues. *The Journal of biological chemistry* **226**, 497-509 (1957).

4 Demberg, T., et al. Strong viremia control in vaccinated macaques does not prevent gradual Th17 cell loss from central memory. Vaccine 29, 6017-6028 (2011).
